# Serum C-peptide assay of patients with hyperglycemic emergencies at the Lagos State University Teaching Hospital (LASUTH), Ikeja

**DOI:** 10.1186/1755-7682-7-50

**Published:** 2014-11-28

**Authors:** Akinyele Taofiq Akinlade, Anthonia Okeoghene Ogbera, Olufemi Adetola Fasanmade, Michael Adeyemi Olamoyegun

**Affiliations:** Department of Medicine, General Hospital, Odan, Lagos Nigeria; Lagos State University Teaching Hospital, Ikeja, Lagos Nigeria; Lagos University Teaching Hospital, Idi-Araba, Lagos Nigeria; Ladoke Akintola University College of Health Sciences/Teaching Hospital, Ogbomosho, Oyo State Nigeria

## Abstract

**Introduction:**

HE are common acute complications of diabetes mellitus (DM) and include diabetic ketoacidosis (DKA), normo-osmolar hyperglycemic state (NHS) and hyperosmolar hyperglycemic state (HHS). They contribute a lot to the mortality and morbidity of DM. The clinical features include dehydration, hyperglycemia, altered mental status and ketosis. The basic mechanism of HE is a reduction in the net effective action of circulating insulin, resulting in hyperglycemia and ketonemia (in DKA) causing osmotic diuresis and electrolytes loss. Infection is a common precipitating factor.

Measurement of serum C-peptide provides an accurate assessment of residual β-cell function and is a marker of insulin secretion in DM patients.

**Aim and objectives:**

To assess the level of pancreatic beta cell function in HE patients, using the serum C-peptide.

**Methodology:**

The biodata and clinical characteristics of the 99 subjects were collated using a questionnaire. All subjects had their serum C-peptide, glucose, electrolytes, urea, creatinine levels, urine ketones determined at admission. Results of statistical analysis were expressed as mean ± standard deviation (SD). A p value <0.05 was regarded statistically significant. Correlation between levels of serum C-peptide and admission blood glucose levels and the duration of DM respectively was done.

**Results:**

The mean age of the subjects was 51 (SD ± 16) years and comparable in both sexes. Mean duration of DM was 6.3 (SD ± 7.1) years, with 35% newly diagnosed at admission.

The types of HE in this study are: DKA (24.7%), NHS (36.1%), and HHS (39.2%). Mean blood glucose in this study was 685 mg/dL, significantly highest in HHS and lowest in NHS. Mean serum C-peptide level was 1.6 ng/dL. It was 0.9 ng/dL in subjects with DKA and NHS while 2.7 ng/dL in HHS (p>0.05).

Main precipitating factors were poor drug compliance, new-onset of DM and infection.

**Conclusion:**

Most (70%) of subjects had poor pancreatic beta cell function, this may be a contributory factor to developing HE. Most subjects with high C-peptide levels had HHS.

**Electronic supplementary material:**

The online version of this article (doi:10.1186/1755-7682-7-50) contains supplementary material, which is available to authorized users.

## Background

### Definition of diabetes mellitus and hyperglycemic emergencies

Diabetes mellitus (DM) is a complex metabolic disorder that has multiple etiologies and is characterized by chronic hyperglycemia as a result of defects in insulin secretion, insulin action, or both. These defects result in the disturbances of carbohydrate, fat and protein metabolism [1].

It is this chronic hyperglycemic state that predisposes diabetic patients to long-term complications such as retinopathy with potential blindness, nephropathy that may lead to renal failure, and/or neuropathy with risk of foot ulcers, amputation, Charcot joints, and features of autonomic dysfunction, including sexual dysfunction [1].

Diabetic patients are also known to be at an increased risk of developing cardiovascular, peripheral vascular and cerebrovascular disease [2].

Hyperglycemic emergencies (HE) are common acute complications of DM and include diabetic ketoacidosis (DKA), normoosmolar hyperglycemic state (NHS) and hyperosmolar hyperglycemic state (HHS). They are life-threatening conditions, even if managed properly and either type can fall anywhere along the disease continuum of diabetic metabolic derangements, only differing in the time of onset, the degree of dehydration, and the severity of ketosis. In view of this, DKA and HHS are not exclusive of type 1 and type 2 DM respectively [3]. These HE contribute a great deal to mortality and morbidity of DM. They are the most serious acute complications of DM and represent the two extremes in the spectrum of diabetic decompensation [4]. It has been established that the basic underlying mechanism for both DKA and HHS is a reduction in the net effective action of circulating insulin [5].

### Classification of diabetes mellitus

The classification of diabetes mellitus (DM) is based mainly on its etiology and pathogenesis. There are four main classes of DM: Type 1 Diabetes Mellitus (T1DM), Type 2 Diabetes Mellitus (T2DM), Gestational Diabetes Mellitus (GDM) and a miscellaneous group referred to as “other specific types” [2]

- Type 1 Diabetes mellitus Type 1 Diabetes Mellitus (T1DM) includes cases due to autoimmune ß cell destruction, which ultimately leads to absolute insulin deficiency and a requirement for exogenous insulin for survival. There are two subtypes: Type 1A, in which there is evidence of autoimmunity characterized by the presence of islet cell autoantibodies, anti-insulin autoantibodies and anti-glutamic acid decarboxylase antibodies. In Type 1B, there is no evidence of autoimmunity, hence it is also called “idiopathic Type 1” [2]. Type 1B constitutes between 5 and 10% of type 1 DM [6, 7]. Persons with T1 DM are insulin dependent and are ketosis prone.
- Type 2 Diabetes mellitus Type 2 Diabetes Mellitus (T2DM) is the most common form of diabetes and is characterized by disorders of insulin action and insulin secretion, which may range from predominantly insulin resistance with relative insulin deficiency to a predominantly secretory defect with or without insulin resistance. This class of diabetes is typically non-insulin dependent and non-ketosis prone [2].
- Gestational Diabetes Mellitus (GDM) This refers to any degree of glucose intolerance with onset or first recognition during pregnancy [2, 8]. Unless known to have diabetes, all women who have been treated as GDM should have an oral glucose tolerance test (OGTT) 6 weeks postpartum in order to reclassify them [9].
- Other specific types of diabetes mellitus These are less common causes of diabetes mellitus, but are those in which the underlying defect or disease process can be identified in a relatively specific manner. They include genetic defects of ß cell function, genetic defects of insulin action, diseases of the exocrine pancreas and endocrinopathies. Others are drug- or chemical- induced and other uncommon immune mediated diabetes and other genetic syndromes [2].

### Epidemiology of diabetes mellitus

In 2007, the International Diabetes Federation (IDF) estimated that approximately 246 million people around the world have DM [10]. This figure is expected to double by the year 2025, representing about 6.3% of the world population. The greater part of this burden is to be borne by the African and Asian continents [10, 11]. The prevalence of DM in Nigeria a decade ago was estimated at 2.2% [12] though reports from some regions of Nigeria have found prevalence rates between 0.9 and 8.3% [13, 14].

In many countries, improved nutrition, better hygiene and the control of communicable diseases have led to increasing longevity that has unmasked many age-related non-communicable diseases including type 2 diabetes and cardiovascular diseases. These formerly uncommon non-communicable diseases have added to many communicable diseases and are now major contributors to ill- health and death. This shifting pattern of diseases known as “epidemiologic transition”, has catapulted type 2 DM from its position as a rare disease at the beginning of the 20^th^ century to its current position as a major global contributor to disability and death and one of the major health challenges of the 21^st^ century [11].

The shift in disease pattern has occurred in developed countries over the past 50 years and is now affecting developing countries. Socioeconomic development, which has resulted in a change of life style from traditional agrarian to modern sedentary lifestyles, consumption of diets rich in fats, and dense in calories all contribute to the current diabetes epidemic [15].

### Morbidity and mortality of DM and hyperglycemic emergencies

The chronic complications of diabetes contribute significantly to its morbidity and mortality. It is a leading cause of end-stage renal disease in the United States, Europe and Japan [16]. In Nigeria, diabetic nephropathy comes after chronic glomerulonephritis and hypertensive nephrosclerosis [17] as a cause of end-stage kidney disease. Diabetes is also a leading cause of new onset blindness in adults aged 20-74 years in the United States where it also accounts for greater than 60% of non-traumatic lower extremities amputations [18].

DM accounts for 5.8% of the total health care costs of citizens of the United States. Hospitalization expenses produced 40.5% of these costs [19].

At the Lagos State University Teaching Hospital (LASUTH), DM accounted for 15% of all medical admissions in 2006, with hyperglycemic emergencies (HE) making up 39.8% of these DM-related admissions and 6.2% of all admissions into the medical wards of LASUTH [20]. Of HE, 88% was DKA while HHS accounted for 12%. A similar study done at the Ahmadu Bello University Teaching Hospital (ABUTH), Zaria in the northern state of Kaduna, Nigeria, documented 40.6% of the patients as having DKA, while 34.4% and 25% had HHS and normo-osmolar nonketotic hyperglycemic state (NNHS) respectively [21]. But reports of studies from developed countries clearly showed HHS as accounting for the majority of HE [22, 23]. In addition, HE have been found to be the most common reasons for DM-related admissions and death throughout the African continent [24–26]. And the incidences of DKA and HHS in these DM-related admissions are close to the Nigerian findings [27].

#### Predictors of mortality in hyperglycemic emergencies

Age, degree of dehydration, hemodynamic instability, underlying precipitating causes, and degree of consciousness are all powerful predictors of a fatal outcome [28, 29]. The hospital admission and mortality rates of patients with diabetic emergencies, such as DKA and HHS, are higher in Black than in White patients with diabetes. In urban Black patients, poor compliance with insulin therapy was the main precipitating cause of acute metabolic decompensation [30]. Although age and degree of hyperosmolarity both influenced mortality rates, only age was found to be an independent predictor of mortality. The mortality rate for DKA remained lower, in most studies, than that for HHS which remained considerably higher, probably because HHS is commoner in the elderly (especially institutionalized) patients who also have significant comorbidities [31].

### Pathogenesis of hyperglycemic emergencies

The basic underlying mechanism for DKA and HHS is a reduction in the net effective action of circulating insulin coupled with a concomitant elevation of counter regulatory hormones, such as glucagon, catecholamines, cortisol, and growth hormone. These hormonal alterations lead to increased hepatic and renal production of glucose as well as impaired glucose utilization in peripheral tissues, which result in hyperglycemia and parallel changes in osmolality of the extracellular space [5]. The combination of insulin deficiency and increased counter- regulatory hormones also lead to release of free fatty acids into the circulation from adipose tissue (lipolysis) and to unrestrained hepatic fatty acid oxidation to ketone bodies (β-hydroxybutyrate and acetoacetate), with resultant ketonemia and metabolic acidosis. In HHS however, while the plasma insulin concentration may be inadequate to facilitate glucose utilization it is adequate to prevent lipolysis and subsequent ketogenesis [32]. Both DKA and HHS are associated with glycosuria, leading to osmotic diuresis with loss of water, sodium, potassium, and other electrolytes [33]. DKA occurs often in patients with type 2 diabetes in response to stressors such as overwhelming infection, infarction of tissue, or other severe illness. DKA occurring without any notable precipitating stress has also been observed [34].

### Clinical and laboratory features of hyperglycemic emergencies

The hallmark of HHS is profound dehydration, marked hyperglycemia, and often some degree of neurologic impairment with mild or no ketosis [4]. The process of HHS usually evolves over several days to weeks, whereas the evolution of the acute DKA episode in type 1 diabetes or even in type 2 diabetes tends to be much shorter. Although the symptoms of poorly controlled diabetes may be present for several days, the metabolic alterations typical of ketoacidosis usually evolve within a short time frame (typically <24 h) [35].

The clinical picture of patients with DKA includes a history of polyuria, polydipsia, weight loss, vomiting, abdominal pain, dehydration, weakness, mental status change, and coma. Physical findings may include poor skin turgor, Kussmaul’s respiration, tachycardia, hypotension, alteration in mental status, shock, fever or hypothermia and ultimately coma. Mental status can vary from full alertness to profound lethargy or coma, with the latter more frequent in HHS [35].

Although infection is a common precipitating factor for both DKA and HHS, patients can be normothermic or even hypothermic primarily because of peripheral vasodilatation [36]. Severe hypothermia, if present, is a poor prognostic sign. Abdominal pain, sometimes mimicking an acute abdomen, is present in 50–75% of DKA cases [37] but usually resolves with correction of hyperglycemia and metabolic acidosis.

Typically, patients presenting with HHS are older and have undiagnosed diabetes or type 2 diabetes managed by diet and/or oral anti-diabetic medication. Such patients often take medications that aggravate the problem, such as a diuretic that causes mild dehydration. Some live alone and may have nobody nearby to communicate their needs to [38]. The most common clinical presentation in patients with HHS is altered sensorium [39]. Other typical presentations include body weakness, visual disturbances, leg cramps. Nausea and vomiting may occur, but are much less frequent than in patients with DKA. A low-grade fever due to infection is often present, and signs of acidosis (Kussmaul breathing, acetone breath) are usually absent. In some patients, focal neurologic signs (hemiparesis, hemianopsia) and seizures (partial motor seizures more common than generalized) may be the dominant clinical features [40].

The degree of neurologic impairment is related directly to the effective serum osmolarity; with coma often occurring once the serum osmolarity is greater than 350 mOsm/kg. Significant overlap between DKA and HHS has been reported in more than one-third of patients [41]. Although most patients with HHS have an admission pH >7.30, a bicarbonate level >20 mEq/l, mild ketonemia may be present.

Initial laboratory findings in patients with HHS include marked elevations in blood glucose (greater than 600 mg/dL [33.3 mmol/L]) and serum osmolarity (greater than 320 mOsm/kg of water), with a pH level greater than 7.30 and mild or absent ketonemia. Fifty percent of the patients will demonstrate a mild anion-gap metabolic acidosis (12–15). Vomiting and use of thiazide diuretics may cause a metabolic alkalosis that could mask the severity of acidosis. Creatinine, blood urea nitrogen (BUN), and hematocrit levels are almost always elevated. HHS produces significant total body losses of many electrolytes.

The majority of patients with HE present with leukocytosis proportional to blood ketone body concentration [31]. However, leukocytosis >25,000 may designate infection and require further evaluation [41]. The admission serum sodium is usually low because of the osmotic flux of water from the intracellular to the extracellular space in the presence of hyperglycemia. An increase in serum sodium concentration in the presence of hyperglycemia indicates a rather profound degree of water loss. Unless the plasma is cleared of chylomicrons, pseudonormoglycemia and pseudohyponatremia may occur in DKA [42]. Serum potassium levels may be elevated or normal. It may be elevated because of an extracellular shift of potassium caused by insulin deficiency, hypertonicity, and acidemia [43]. Patients with low normal or low serum potassium concentration on admission have severe total-body potassium deficiency and require very careful cardiac monitoring and more vigorous potassium replacement. This is because treatment lowers potassium further and can provoke cardiac dysrhythmia. It has been established that the total body deficit of sodium and potassium might be as high as 500–700 mEq [44]. Studies on serum osmolality and mental alteration have established a positive linear relationship between osmolality and mental obtundation [36].

Finally, abnormal acetoacetate levels may falsely elevate serum creatinine if the clinical laboratory uses a colorometric method for the creatinine assay [45].

#### Precipitating factors of hyperglycemic emergencies

The two most common precipitating factors in the development of DKA or HHS are inadequate or inappropriate insulin therapy and infection. Infections are the leading cause of HHS (57.1 percent); the most common infection is pneumonia, often gram negative, followed by urinary tract infection and sepsis [31, 32]. Other precipitating factors include pancreatitis, myocardial infarction, pulmonary embolism, cerebrovascular accident, and drugs [36, 46] such as corticosteroids, thiazides, and sympathomimetic agents (e.g., dobutamine and terbutaline) [47] and second-generation antipsychotics agents [39]. In addition, new-onset type 1 diabetes or discontinuation of insulin in established type 1 diabetes (due to fear of weight gain, hypoglycemia, rebellion against constituted authority, and the stress of chronic disease) commonly leads to the development of DKA (21 percent). Psychological problems in these young patients complicated by eating disorders may also contribute in 20% of recurrent ketoacidosis [48]. Some studies indicate that over half of newly diagnosed adult African-American and Hispanic subjects with unprovoked DKA have type 2 diabetes [3]. In such patients, clinical and metabolic features of type 2 diabetes include a high rate of obesity, a strong family history of diabetes, a measurable pancreatic insulin reserve, low prevalence of autoimmune markers of ß-cell destruction, and the ability to discontinue insulin therapy during follow-up [49].

### Pancreatic ß cell dysfunction in type 2 diabetes mellitus

Two major metabolic defects characterize type 2 DM: insulin resistance and an insulin secretory defect that is not autoimmune-mediated [50]. The progression from impaired glucose tolerance (IGT) to early T2DM is marked by a decrease in pancreatic ß cell function and thus a decline in insulin secretion. It is the failure over time of the ß cell to compensate for insulin resistance with hyperinsulinemia that marks the beginning of T2DM. Eventually, exhaustion of the pancreatic ß cells occur leading to a progressive, then an absolute insulin deficiency [50]. Recognized ß-cell abnormalities in type 2 diabetes include dysrhythmic pulsatile insulin secretion, defective glucose potentiation of responses to non-glucose insulin secretagogues, increased plasma pro-insulin to insulin ratio, and accumulation of islet amyloid polypeptide [51]. Some of these abnormalities may be genetically mediated occurring early and preceding the onset of hyperglycaemia [52, 53]. Other factors that contribute to the insulin secretory defect include chronic glucotoxicity (damage to the ß cell from chronic exposure to hyperglycemia) and lipotoxicity (ß cell damage resulting from chronic exposure to elevated free fatty acids) [31].

#### Serum c-peptide levels

The measurement of serum C-peptide provides an accurate assessment of residual beta-cell function and thus has been widely used as a marker of insulin secretion in patients with diabetes [45, 54].

The importance of measuring C-peptide has increased significantly in recent years with the evidence from the Diabetes Control and Complications Trial (DCCT) that higher C-peptide concentrations are associated with improved glycosylated hemoglobin (HbA1c) concentrations, less hypoglycemia, and less retinopathy and nephropathy [55]. Thus C-peptide level can serve as an effective index for selecting a diabetic treatment. It has been shown that basal serum C-peptide levels are useful indicators for determining the proper timing to introduce the intensive insulin therapy into DM patients [56]. They were, also, of greater value in identifying patients suitable for oral therapy than any single clinical criterion, and thus may help in identifying insulin-treated diabetic patients who may be treated with oral therapy without deterioration in metabolic control [57].

### Relevance and benefit of the study

This study will help draw the managing physicians’ attention to the need for proper differentiation of the types of HE they are dealing with based on established criteria. The serum C-peptide assay will help determine the appropriate medications for the patients in the study following recovery from HE, by providing an insight into the level of residual pancreatic beta cell function. This will translate into better patient management and improved outcome.

#### Aims and objectives

##### Aim

To assess the level of pancreatic β cell function in patients with hyperglycemic emergencies.

##### General objectives

1. To describe the types of hyperglycemic emergencies in LASUTH
2. To describe the clinical and biochemical characteristics of patients with hyperglycemic emergency managed at LASUTH.

##### Specific objectives

1. To determine the serum C-peptide levels of patients admitted for hyperglycemic emergencies (HE) in LASUTH with a view to determining the level of pancreatic beta cell function in patients with hyperglycemic emergency.
2. To predict which of the patients in this study will respond to oral hypoglycemic agents or will require continued insulin use after recovery from hyperglycemic emergency.

## Literature review

### The endocrine pancreas

#### Embryology and anatomy

**Figure 1:**
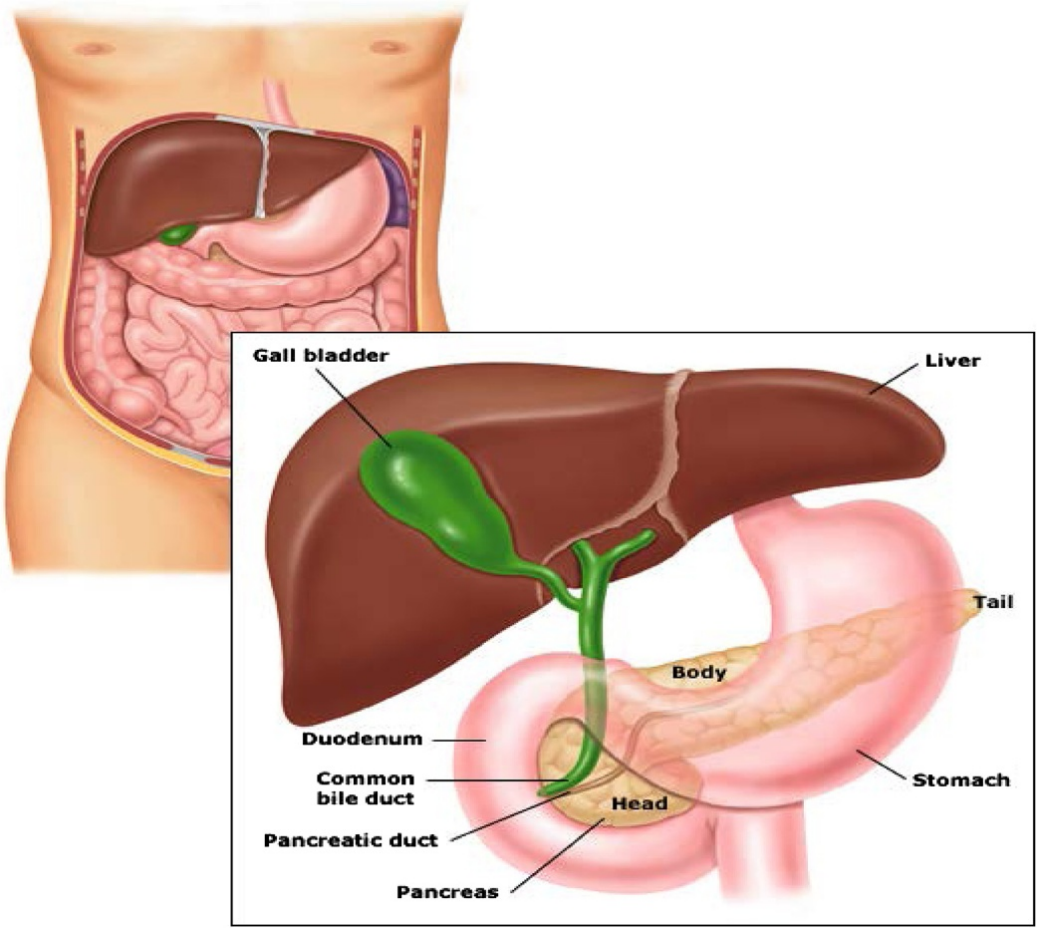
**Structure of the pancreas and its anatomical relationships (courtesy** http://www.livelongagewell.com **).**

The pancreas (Figure 1) is a pale grayish-yellow retroperitoneal organ surrounded by a thin connective tissue capsule. It weighs 75-100 g, is approximately 15-20 cm in length and extends across the posterior abdominal wall from the second part of the duodenum to the spleen. It is divided into the head and uncinate process, the neck, the body, and the tail. The duodenum encircles its head. The body, which forms the main bulk of the organ, ends in a tail that lies in contact with the spleen. The main pancreatic duct (duct of Wirsung) is 2-3.5 cm wide, runs in the center of the pancreas, and drains the body, tail and uncinate process. The lesser duct (duct of Santorini) usually drains the head, communicates with the duct of Wirsung, and drains separately via a minor papilla located 2 cm proximal to the ampulla of Vater. The common bile duct is found posteriorly in the pancreatic head and joins the main pancreatic duct before draining into the ampulla [58, 59].

The pancreas originates as dorsal and ventral pancreatic buds from the primitive endoderm at about the fifth week of gestation. The former gives rise to the superior head, neck, body, and tail, whereas the latter forms the inferior head and the uncinate process. The ventral duct fuses with the dorsal bud to form the duct of Wirsung, and the proximal portion of the duct forms the duct of Santorini. In 10% of individuals, the ducts fail to communicate, resulting in pancreatic divisum, where the entire pancreas is drained by the lesser duct [59].

Paul Langerhans discovered a group of cells in the pancreas arranged in islets, known as the islet of Langerhans in 1869 and this preceded the discovery of “pancreatic-diabetes” 20 years later by the experiments of Mooring and Minkowski [60].

The glandular tissue of the pancreas (both exocrine and endocrine) develops by budding and branching of the primordial epithelial cell cords. The islets originate from specialized buds of the same epithelial cords. During embryonic life, the glucagon producing α cells are generally the first to develop and the pancreatic polypeptide (pp) cells the last. The α cells can be recognized morphologically at about 9 weeks of gestation, the delta cells at 10 weeks and β cells at 11 weeks. Hormones begin to be produced as from 3 weeks after these cells are morphologically recognizable [61].

#### Histology of the pancreas

**Figure 2:**
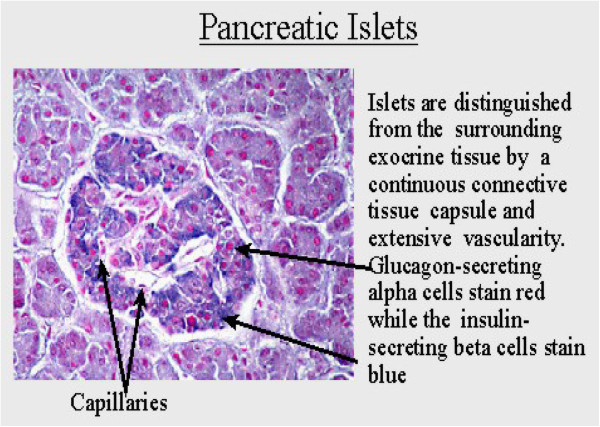
**Histological section of the pancreas (courtesy** http://www.webanatomy.net **).**

The normal adult pancreas contains around 1 million islets cells constituting about 2 - 3% of the total gland mass. The different islet cell types can be distinguished histologically and by various histochemical techniques (Figure 2). On routine histologic staining with hematoxylin and eosin, the islet cells of Langerhans can be readily identified as they stain less intensely than the surrounding parenchyma. In humans, there are four different histologically identifiable cell types: α, β, ∂, and the pp (pancreatic polypeptide) cells. The proportions of the different islet cell types vary with age from 45:32:22:1% (for β: ∂: α: pp cells, respectively) in neonates to 68: 10: 20: 2% in adults [61].

β cells are the most numerous islet cell types and their identity as source of insulin was established in 1938 when the β cells of dogs with diabetes (induced by anterior pituitary extract rich in growth hormone) were found to be extensively deregulated or destroyed. They characteristically stain positive with aldehyde fuchsine and form the homocellular medulla [61].

The α cells lie in the islet cortex together with the other non- β cells and are more numerous in the tail and body of the pancreas, sparse in the head. Other minor cell types with secretory granules in the islets include D_1_ cells, which are similar to the pp cells but are not immunoreactive for pancreatic polypeptide and also the rare p cell types have been identified [61].

#### Hormones of the endocrine pancreas

Glucagon is the main product of the α -cells but other peptides derived from the pre-pro-glucagon precursor are also synthesized and include glicentin (gut glucagon) and the glucagon–like peptides (GLP–1 and GLP–2) [61]. Glucagon is believed to act exclusively on the liver under physiologic conditions. It activates glycogenolysis, and to some extent gluconeogenesis, and increases hepatic glucose production within minutes.

The δ cells produce somatostatin, which is a 14–amino acid peptide that suppresses growth hormone release and is produced by neurons, and endocrine cells in many sites throughout the body. Its action includes suppression of the secretion of both insulin and glucagon possibly through both endocrine and paracrine effects.

The pp cells, originally termed f cells produce pancreatic polypeptide, a 36 – amino acid peptide, which is expressed exclusively in the pancreas and the exact function is unknown [61].

Other bioactive polypeptides, which have been localized to the pancreatic islets, include glutamic acid decarboxylase (GAD), which is expressed by the β cells and is thought to be one of the auto-antigens responsible for inciting the autoimmune damage leading to type 1 diabetes, and the islet amyloid polypeptide (IAP), which has been found to contribute to the pathogenesis of type 2 DM [61].

### Biosynthesis and structure of insulin

#### Biosynthesis of insulin

Insulin is the major hormonal regulator of glucose metabolism. It was first isolated from pancreatic tissue in 1921 by Banting and Best [62]. In humans, the gene encoding pre-pro-insulin that is the ultimate precursor of insulin is located on the short arm of chromosome 11 [62].

**Figure 3:**
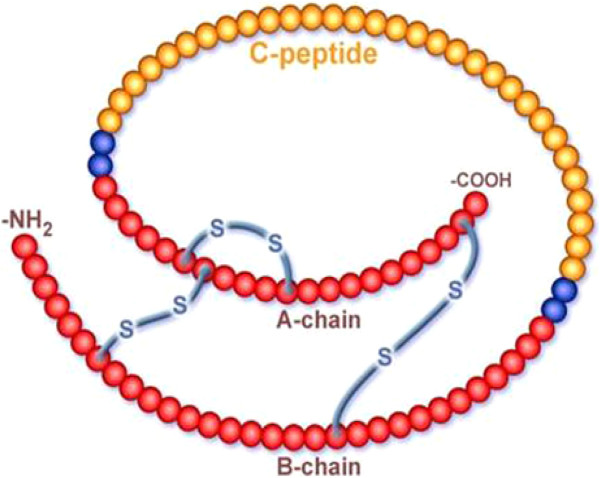
**Structure of pro insulin showing C-peptide and the A and B chains of insulin (courtesy** http://www.cebix.com/media/images **).**

Pre-pro-insulin is cleaved by proteolytic enzymes into pro-insulin. Pro-insulin is a 9-KDA peptide, containing A and B chains of insulin which contain, respectively, 21 and 30 amino acid residues joined by the C-peptide which has 30-35 amino acids (Figure 3). It undergoes proteolytic cleavage by endopeptidases (pro-hormone convertase and carboxypeptidase) with liberation of cleavage dipeptides, Arg [31] – Arg [32], Lys [62] – Arg [63], so yielding the split products and ultimately insulin and C-peptide [62].

Insulin and C-peptide are stored together in secretory granules and ultimately released in equimolar amounts. 95% of the hormone product is secreted as insulin and less than 5% as unconverted pro-insulin (Figure 3). This pathway represents the regulated pathway of insulin secretion. An alternative constitutive pathway operates that omits the conversion and packaging stages and allows the release of insulin from vesicles derived from the endoplasmic reticulum. The major product of this pathway is pro-insulin and is released in large amounts in insulinomas. Measurements of pro-insulin in evaluation of hypoglycaemia are clinically relevant; plasma pro-insulin concentration is elevated irrespective of the prevailing blood glucose concentrations in patients with insulinomas while the concentration of insulin and C-peptide may be within the normal range [62].

#### Insulin secretion

Insulin is secreted from the secretory granules in the pancreatic β-cell by exocytosis in which the granule membrane and the plasma membrane fuse together and the granule’s contents diffuse across the capillary membrane into the blood stream. The major physiological determinant of insulin secretion in mammals is the blood glucose concentration. It acts with other nutrients as initiators of the insulin secretory process [62]. Pancreatic β cells are acutely sensitive to small changes in extracellular glucose concentrations within a narrow physiological range. Glucose is transported into β cells via the glucose transporter (GLUT)–2 that allows rapid equilibration of extracellular and intracellular glucose concentrations within seconds. The other glucose transporters also play a role in the transport of glucose into cells and tissues. GLUT 1 is present in all tissues, has high affinity for glucose and mediates basal glucose uptake. It is therefore able to transport glucose at relatively low concentration as found in the basal state. GLUT 3 is also found in all tissues and is the major glucose transporter on the neuronal surface. It has a very high affinity for glucose and is responsible for transferring glucose into neuronal cells. In contrast, GLUT 2 has a very low affinity for glucose and seems to act as a transporter only when plasma glucose levels are relatively high, such as following a meal. It is a major transporter of glucose in hepatic, intestinal and renal tubular cells. GLUT 4 is found in two major insulin target tissues, skeletal muscle and adipose tissue. It facilitates the transport of glucose into these storage tissues after a meal.

In the β cell, glucose is phosphorylated by glucokinase, which acts as the glucose sensor coupling insulin to the prevailing glucose level [63].

The characteristic dose-response curve of insulin is sigmoidal in shape and it is determined primarily by the regulatory activity of glucokinase. Concentrations of glucose below 5 mmol/L do not affect rates of insulin release and the largest increase in secretory rates occurs at extracellular glucose levels between 5–17 mmol/L. An acute first phase insulin secretion occurs lasting a few minutes followed by a sustained second phase of secretion, which persists for the duration of the high glucose stimulus [64].

In the β cell, glucose must be metabolized via glycolysis to produce adenosine triphosphate (ATP), which closes specific ATP– sensitive potassium channels in the β cell membrane. This prevents potassium ions from leaving the β cell, thereby causing membrane depolarization. The membrane depolarization causes the voltage–gated calcium channels in the membrane to open, leading to the influx of calcium into the beta cell membrane. The increase in cytosolic calcium causes the release of insulin from the beta cells [64].

Several amino acids are effective insulin secretagogues in vivo and in vitro. Most require glucose but some like leucine, lysine and arginine do not. Leucine stimulates insulin secretion in a mechanism similar to that of glucose [65].

Hormones also affect the secretion of insulin either in an autocrine or paracrine manner or by reaching the β cells via the circulation. Secretagogues include glucagon, acetylcholine, cholecystokinin and pituitary adenylate cyclase–activating polypeptide while inhibitors include somatostatin, pancreastatin, adrenaline and norepinephrine. Cholinergic, adrenergic and peptidergic neurons innervate the islet cells and release of neurotransmitters from these autonomic nerve endings can modulate insulin secretion [65].

#### Structure of insulin

The primary amino acid sequence of porcine insulin was first reported by Frederick Sanger *et al.*[66] about 50 years ago and Dorothy Hodgkin *et al.*[67] used x-ray diffraction to determine the three dimensional structure of the insulin hexamer.

The insulin molecule consists of two polypeptide chains designated A and B linked by two disulphide bridges with a molecular weight of about 6000 Daltons. The A chain also contains an intrachain disulphide bridge linking residues 6 and 11. The A chain consists of 21 amino acid residues and the B chain consists of 30 amino acid residues [67] (Figure 3).

Human insulin differs from porcine insulin in the last residue of the B chain where threonine is found in humans, and alanine in pig. It differs from bovine insulin due to the substitution of threonine and isoleucine in humans with alanine and valine in ox at positions 8 and 10 respectively in the A chain. These differences have significant implications in the pharmacokinetic properties of human and animal insulins. Bovine insulin has a slightly altered solubility and crystallization behavior and the single amino acid difference between human and porcine insulin makes it less lipophilic and absorbed slightly more rapidly [68].

#### Physiologic actions of insulin

Insulin has many physiologic effects, which can be divided into intermediate effects, which regulate intermediary metabolism, and chronic effects, which stimulate growth and proliferation [66].

The intermediary effects involve the regulation of glucose, lipid and protein metabolism. It regulates glucose metabolism by inhibiting glucose production by the liver and by stimulating glucose uptake, particularly in skeletal muscle. Actions on lipid metabolism include the promotion of the synthesis of lipids and the inhibition of lipolysis in adipose tissue. When intact, these actions lead to lowering of serum glucose, triglycerides and non-esterified fatty-acid (NEFA) concentrations and to a decrease in lipoprotein lipase activity in adipose tissue [69].

The intracellular actions of insulin are mediated through two different cascades of protein intermediates. One pathway transmits the insulin signal through insulin receptor substrate proteins and phosphosphatidyl inositol 3-kinase (PI 3-kinase) to a series of intracellular proteins, which culminate in increasing glucose transport and utilization, and decreasing adipose tissue lipolysis as well as regulating other aspects of intermediary metabolism. The other pathway involves activation of the mitogen-activated protein (MAP) kinase–pathway, which increases growth and the mitogenic processes [69].

Insulin also induces slow vasodilatation and diminishes stiffness of large arteries, which are thought to protect against the development of hypertension. It acts centrally by regulating the tone of the autonomic nervous system stimulating the sympathetic outflow to the cardiovascular system and other tissue. Its effects on electrolyte balance include enhanced sodium and uric acid re-absorption in the kidneys, and stimulation of potassium uptake into peripheral tissues [69].

### Connecting (C-) peptide

Connecting peptide (C-peptide) consists of 31 amino acid residues with a molecular weight of 3021 Daltons (Figure 3). It is co-secreted with insulin by the pancreatic β cells as a by-product of the enzymatic cleavage of pro-insulin to insulin. C-peptide and insulin are secreted into the portal circulation in equimolar concentration. It is called C-peptide because it connects the A and B chains of insulin in the pro-insulin molecule. While the liver clears a significant portion of insulin (50-60%) in a first pass; C-peptide does not undergo hepatic extraction and has constant peripheral clearance at various plasma glucose concentrations. It is for these reasons that plasma C-peptide concentrations may reflect pancreatic insulin secretion more reliably than the level of insulin itself [70, 71]. The standard radioimmunoassay for measuring insulin concentrations is also unable to distinguish between endogenous and exogenous insulin, making it ineffective as a measure of endogenous β cell reserve in insulin-treated diabetic patients. In addition, anti-insulin antibodies that may be present in insulin-treated patients interfere with the insulin radioimmunoassay, making insulin measurement in insulin-treated patients inaccurate. Conventional insulin radioimmunoassays are also unable to distinguish between levels of circulating proinsulin and true levels of circulating insulin. Also (and unlike insulin), C-peptide has no known physiological function.

It is excreted exclusively by the kidney and its plasma half-life of ~30 minutes contrasts sharply with the short plasma half-life of insulin (~4 minutes). C-peptide assays are widely available in which the relative molar cross-reactivity of pro-insulin and pro-insulin conversion products compared with C-peptide is ≅ 10% and therefore contribute a negligible amount to total C-peptide immunoreactivity [70].

While no local studies are currently available on the measurement of serum C-peptide in patients with hyperglycemic emergency, it is well known that measurement of serum C-peptide provides an accurate assessment of residual beta-cell function and thus has been widely used as a marker of insulin secretion in patients with diabetes [45].

Measurement of C-peptide under standardized conditions provides a sensitive, well accepted, and clinically validated assessment of ß-cell function [70].

Some studies have also suggested that C-peptide is biologically active [72] and may play a role in preventing and possibly reversing some chronic complications of type 1 diabetes [73, 74].

The importance of measuring C-peptide has increased significantly in recent years with the evidence from the Diabetes Control and Complications Trial (DCCT) that higher C-peptide concentrations are associated with improved HbA1c concentrations, less hypoglycemia, and less retinopathy and nephropathy [55]. Thus C-peptide level can serve as an effective index for selecting a diabetic treatment. It has been shown that basal serum C-peptide levels are useful indicators for determining the proper timing to introduce the intensive insulin therapy into DM patients [56]. They were, also, of greater value in identifying patients suitable for oral therapy than any single clinical criterion, and thus may help in identifying insulin-treated diabetic patients who may be treated with oral therapy without deterioration in metabolic control [57].

Ethnic differences may have a role to play in determining the development of beta-cell depletion in type 2 DM patients. Black South African type 2 [75] diabetic subjects have been found to be more likely than their African-American counterparts to develop depletion of beta-cell function. Black South African subjects categorized as having type 2 DM presenting with DKA may thus not have the relatively good insulin reserve shown in African-American patients, but are more likely to be insulin-deficient at the time of presentation, or present with a late-onset form of auto-immune diabetes (LADA). In a study, approximately a quarter of Black patients presenting with DKA do so with a relatively good beta-cell reserve [76]. This is in contrast to a Nigerian study of type 2 DM patients who showed poor pancreatic beta cell response to changing levels of plasma glucose [77]. This finding in this Nigerian study is in consonance with the increasing evidence from several cross-sectional and longitudinal studies examining β cell function and insulin sensitivity which is suggesting that impaired β cell function is the primary underlying, possibly genetic defect [78–80].

### Pancreatic ß cell defects in type 2 diabetes mellitus

For many years, it had been controversial whether impaired ß cell function or tissue insulin resistance is the underlying pathogenetic element in type 2 diabetes. Until quite recently, it was generally thought that insulin resistance preceded ß cell dysfunction and was the primary genetic factor while ß cell dysfunction was a late phenomenon due to exhaustion after years of compensatory hypersecretion [81].

However, during the past several years, the accumulation of evidence from several studies examining ß cell function and tissue insulin sensitivity both cross-sectionally and longitudinally has swung the pendulum over to the concept that impaired ß cell function is the primary underlying, possibly genetic defect [51, 76, 79].

By the time type 2 diabetes is diagnosed, defects in ß cell function are quite obvious. In the UKPDS, evaluation of ß cell function using the homeostasis model assessment (HOMA), indicated that ß cell function was already reduced by 50% at diagnosis and that there was subsequent further deterioration, regardless of therapy [82]. Observations of impaired ß cell function in studies of first degree relatives of individuals with type 2 DM with normal glucose tolerance and absence of insulin resistance provide further evidence that impaired ß cell function is the primary genetic defect in type 2 diabetes [76].

Abnormalities of insulin secretion in persons with overt type 2 diabetes include reduced or absent first-phase responses to intravenous glucose, delayed and blunted secretory responses to ingestion of a mixed meal, alteration in rapid pulses and ultradian oscillation of insulin secretion and an increase in the plasma concentration of pro-insulin [76].

Persons with impaired glucose tolerance also demonstrate ß cell secretory defects including reduced early insulin secretory response to oral glucose, reduced ability of the ß cell to compensate for the degree of insulin resistance as evidenced by reduced first-phase insulin secretory responses in relation to the degree of insulin resistance and defects in oscillatory insulin secretion [79].

A number of different hypotheses have been advanced as explanations for the development of ß cell dysfunction in type 2 diabetes [51]. These include:

(A) ß cell exhaustion
  Pancreatic ß cell exhaustion is thought to result from an increased secretory demand arising from insulin resistance. Under normal circumstances, insulin resistance increases the secretory function of the ß cell. This increase in the need for insulin biosynthesis and release has led to the suggestion that over a period of time, the increasing demand associated with increasing resistance will result in exhaustion of the ß cell so that it ultimately fails [50]. This exhaustion is, however, thought to be a genetically programmed ß cell abnormality associated with an inability of the normal ß cell to adapt to insulin resistance with increased secretory demand thus uncovering a preexisting defect in ß cell function. On the other hand, the ß cells in individuals without such a genetic lesion would adapt and prevent the development of hyperglyceamia [78].
(B) Desensitization of the ß cells
  Desensitization of the ß cell occurs from chronic glucotoxicity and lipotoxicity. Chronic glucotoxicity leads to the deposition of advanced glycation end-products in the ß cells [83]. Acute and prolonged hyperglycemia also cause down regulation of glucose transporters and oxidative stress–induced alteration of transcription factors [84]. Triglyceride accumulation within the ß cell is also thought to contribute to the ß cell dysfunction [85].
  Islet amyloid deposition is present in 90% of patients with type 2 diabetes mellitus (derived from islet amyloid polypeptide (amylin) which is co-secreted with insulin from beta cells) and its accumulation is associated with progressive reduction in insulin secretion and glucose tolerance [86].
(C) Reduction In ß Cell Mass
  This occurs in about 20–30% of individuals with type 2 DM and is thought to result from programmed cell death (apoptosis) resulting from lipotoxicity, glucotoxicity and amyloid deposition.
  Other tissue factors from adipose tissue contributing to impaired β-cell function include tumor necrosis factor (TNF)-α, resistin and leptin [85].

### Assessment of pancreatic ß - cell function

Several methods exist for the estimation of the insulin secretory reserve and can be grouped into:

I. Basal methods
II. Dynamic methods
III. Steady state methods.

#### Basal methods of assessing pancreatic ß cell function

Blood glucose is related to circulating c-peptide and insulin levels. Basal measurements of glucose, insulin and C-peptide levels have been used to estimate the degree of pancreatic β cell function in patients with types 1 and 2 DM [86].

The widely used Homeostasis model assessment (HOMA) model developed by Mathews *et al.*[86] uses fasting measurements of blood glucose and insulin concentrations to calculate an index of ß cell function (HOMA%B) and insulin resistance (HOMA-IR).

The principle of HOMA is that blood glucose and insulin concentrations are related by the feedback of glucose on the ß- cells to increase insulin secretion. Therefore, for a given level of blood glucose, prevailing insulin levels reflect both insulin sensitivity and ß cell function. This relationship is however complex [86]. The model assumes that normal weight subjects less than 35 years have 100% ß cell function [86]. HOMA1, the original model contains nonlinear empirical equations, which are widely used. The HOMA index of insulin secretion (HOMA%B) is calculated as follows:

HOMA%B = (20 Χ FPI)/(FPG - 3.5) Where FPI is fasting plasma insulin concentration (mU/L) and FPG is fasting plasma glucose (mmol/L). Normal HOMA%B is 100% [87].

HOMA2, the updated model or the computer model also has nonlinear equations and is better used when HOMA is being compared with other models. The HOMA model has been found to correlate well with other methods of assessment of insulin secretion [87].

Basal methods are cheap, simple and quick but results are more accurate with more than two insulin samples and can be confounded by exogenous insulin [86].

#### Dynamic methods of assessing pancreatic ß cell function

Direct measurements of plasma insulin and C-peptide concentrations or following pancreatic ß cell stimulation remain one of the most widely used methods of assessing ß cell function [88]. Insulin and C-peptide release can be stimulated by nutrients such as glucose (given orally or intravenously) amino acids like leucine and arginine and by drugs. Direct C-peptide measurements are often preferred to insulin measurements because of lack of hepatic extraction, slower metabolic clearance rates and lack of cross reactivity with antibodies to insulin [88].

Determination of fasting or basal C-peptide (FCP) and stimulated c-peptide (with glucose or glucagon) levels have been used to determine beta cell secretory activity. The C-peptide response to glucagon correlates well with the stimulated response to mixed meals or other stimulus commonly used to characterize endogenous insulin secretion and has the advantage of a shorter duration and simple standardization [88]. Peak serum C-peptide concentration at 6 minutes after bolus injection of 1 mg glucagon is generally between 1-4 ng/ml in normal and type 2 diabetic subjects (‘C-peptide positive’). However, it is ≤0.6 ng/ml in C-peptide negative states such as in established type 1 diabetes [86].

Several studies have used these tests (fasting and post glucagon C-peptide estimations) to determine whether ß cell function shows concordance with the clinical classification of diabetes into type 1 DM and type 2 DM. Although different thresholds for FCP and post-glucagon C-peptide (PGCP) have been used, most studies conclude that there is a high degree of concordance, ranging between 86 and 89% between C-peptide levels and type of diabetes. Some have suggested that the post-glucagon level is the better discriminator. FCP and PGCP have also been found to predict the need for insulin treatment in type 2 diabetes [89].

The standard oral glucose tolerance test (OGTT) with measurements of first phase and second phase insulin release also provides a dynamic measure of ß-cell function [90].

In addition, the frequently sampled intravenous glucose tolerance test (FSIVGTT) also provides a convenient index of insulin secretion. First phase and second phase insulin responses are determined from the insulin peaks as measures of insulin secretion [86].

#### Steady state methods of assessing insulin secretion

Steady state techniques involve the continuous intravenous infusion of insulin with or without glucose until nearly constant plasma glucose concentration is reached [86].

The hyperglycemic clamp is described as the gold standard for the assessment of ß cell function [91]. The clamp is used to measure insulin secretion under steady state condition of controlled hyperglycemia of about 10 mmol/L. The insulin response is biphasic, with an early peak, followed by a sustained rise. Indices of glucose-stimulated insulin secretion include the amplitude of the early peak, the final plasma insulin concentration achieved or the absolute rise from baseline. These data are expressed as percentage of levels in normal subjects [86]. This method is however laborious and is unsuitable for epidemiological studies [91].

Other measures of ß cell function include measurement of circulating insulin precursors. Increased fasting plasma pro-insulin has been described as an indication of ß cell stress with defective posttranslational processing and or accelerated granule secretion before processing is completed [92]. The ß score is relatively new and is used in the assessment of ß cell function after islet cell transplantation [93].

### African and nigerian studies on pancreatic ß-cell function

There are no readily available studies on the level of serum C-peptide and insulin in patients with hyperglycemic emergencies in Nigeria and Africa. However, several studies in Africa, including Nigeria have investigated ß cell function and insulin resistance in subjects with type 2 DM [32, 89, 94–102].

#### Studies on pancreatic ß - cell function

ß cell function studies have been employed in the etiologic classification of diabetes and evaluation of treatment [32, 94–96].

Bella *et al.*[94] assessed the insulin secretory capacity in 3 groups of Nigerians with diabetes using fasting C-peptide and C-peptide levels post-stimulation with oral glucose. The first group consisted of eight subjects with past history of ketoacidosis and were treated with insulin. The second group of six subjects had no history of ketoacidosis but required insulin for the normalization of blood sugar while the third group were eight non- insulin dependent subjects with no history of ketoacidosis and were treated with diet and oral glucose lowering agents. Non-diabetic controls were also included in the study. They found a relationship between C- peptide levels and groups of diabetes. The results of the C-peptide levels in groups 1 and 3 were similar to those of type 1 and 2 diabetics in Caucasians. The group 2 subjects appeared to the authors to have a different form of diabetes that needed further characterization [94].

In a similar study, Abutu [95] assessed ß cell function in a group of young insulin treated Nigerians with diabetes. Fasting and post-glucagon stimulation plasma C-peptide levels were measured. Using recommended values for fasting and post-glucagon stimulated C- peptide levels in type 1 diabetes, 10 (21%) of the subjects studied were classified as insulin-dependent while the remaining 36 (78%) were classified as insulin-treated [95].

Sriraj *et al.*[89] studied fasting and post-glucagon stimulated C-peptide levels in a group of Ethiopians with diabetes. The study included 56 subjects with type 1 DM, 97 subjects with type 2 DM and 50 control subjects. Eighty-six of the total subjects had glucagon stimulation. The results of their study showed that mean fasting; post-glucagon stimulation and increment in C-peptide levels in subjects with type 1 DM were lower than those with type 2 DM or control subjects. In this study, six of the subjects clinically classified as type 2 DM were found to have C-peptide indices similar to type 1 DM subjects. They also had low body mass index (BMI) and were on insulin. This group possibly represented a clinical misclassification or subjects with latent autoimmune diabetes of the adult. Further islet cell antibodies testing in these six patients showed 2 were positive for glutamic acid decarboxylase antibodies [89]. The authors also concluded that C-peptide levels might be useful in identifying subjects with type 2 DM who require insulin therapy.

It is noteworthy that in the study in Ethiopia by Sriraj *et al.*[89], fasting C-peptide levels were higher in subjects with type 2 DM than control subjects. This observation was contrary to the similarity in fasting C-peptide levels in type 2 DM subjects and control group as noted by Bella *et al.* in Nigerians.

Oli *et al.*[32] studied the association between ß cell function and response to treatment in Nigerians with type 2 DM. In that prospective study, 116 subjects were studied. Fasting and post-glucagon stimulation C-peptide indices were determined at baseline. Following a 9- month period, subjects were classified based on glycemic control using fasting plasma glucose at the end of the study and treatment modalities. The group with poor control and that likely required insulin at the end of the study had lower fasting and post-glucagon stimulation C-peptide at baseline. The authors concluded that post-glucagon C-peptide estimation was a good predictor of Nigerians with type 2 diabetes who will likely require insulin for control and a single estimation of post-glucagon C-peptide levels may be a useful guide in predicting likelihood of the need for insulin [32].

In a recent study, Bakari *et al.*[96] studied pancreatic ß cell function in Northern Nigerians with type 2 DM using HOMA technique. In this cross sectional study, 40 subjects with type 2 DM and 36 controls were evaluated. They found that, subjects with type 2 DM had significantly lower pancreatic ß cell function compared with control subjects [96].

Apart from the glucagon stimulation test and HOMA, the IVGTT method has been used to determine pancreatic ß cell function in Nigerians.

Osa [97] determined the pancreatic ß cell function in first-degree relatives of patients with type 1 DM using IVGTT and measuring glucose disposal rates. The glucose disposal rate of 17.2% of the first-degree relatives was less than 10% of the glucose disposal rate of control subjects. They were thus considered to have abnormal glucose tolerance. The abnormal intravenous glucose tolerance indirectly signified abnormal beta cell function. It was concluded that the familial predilection for the development of overt disease is preceded by subclinical glucose tolerance [97].

## Subjects, materials and methods

### Study site

The study was carried out at the Medical Emergency and Wards of the Department of Medicine of the Lagos State University Teaching Hospital (LASUTH), Ikeja, Lagos. The Lagos State University Teaching Hospital is an urban tertiary health centre in Lagos state, Western Nigeria. It is a major referral center serving the whole of Lagos State (3345 sq km/1292 sq m), which is a major point of entry into Nigeria from different parts of the world and the economic nerve centre of Nigeria. LASUTH’s Department of Medicine has five adult medical wards- (three Male and two Female wards). The total bed capacity in the five wards is 93 (40 beds for females and 53 for males).

The medical emergency is a 25-bed facility where new patients are stabilized before being transferred to the wards after 48 hours of admission. Bed occupancy is 100% at most times.

### Subjects

Ninety-seven consecutively admitted patients for hyperglycemic emergency and who met the inclusion criteria for the study were recruited for the study. All the patients had their serum C-peptide levels assessed, in addition to serum electrolyte, urea and creatinine and urinalysis for ketones.

Each patient’s bio-data and clinical characteristics were collated using an investigator-administered questionnaire (see Additional file 1).

The purpose of the study, details of the procedure and the significance of the study to improved practice and management of DM were explained to the patients.

#### Inclusion criteria

1. All patients with diabetes mellitus admitted into the medical wards during the study period and whose random/fasting blood sugar is ≥250 mg/dl, have significant ketonuria, high/normal anion gap, high serum osmolality and with/without an altered sensorium.
2. Patients aged 15 years and above.

#### Exclusion criteria

1. patients with clinical features of stroke or of alcohol/drug-induced coma
2. patients with rebound hyperglycemia following treatment for hypoglycemic episodes
3. pregnant women
4. patients with features of or known to have secondary diabetes mellitus.

### Consent and ethical clearance

All patients gave informed written consent after due explanation either personally or through their relatives (see Additional file 2). The Research and Ethics Committee of the Lagos State University Teaching Hospital approved the study proposal (Additional file 3). The study was conducted between 1^st^ January and 15^th^ June 2009.

### Methods

#### Clinical characteristics

##### Biodata and clinical history

An investigator-administered questionnaire (see Additional file 1) was used to collate each subject’s data such as age at the last birthday and the gender.

The clinical history such as prior history and duration of DM, types of treatment used and compliance, family history (in first-degree relative) of DM, prior history and duration of systemic hypertension, as well as the presence or absence of symptoms such as polyuria, polydipsia, polyphagia and weight loss were also recorded in the questionaire.

The subjects were classified into 3 age-groups in order to allow for easy data analysis:

1. Young age: subjects whose ages were less than 40 years.
2. Middle age: subjects whose ages were between 40 and 60 years
3. Old age: subjects older than 60 years of age.

The duration of DM was classified into 3 groups to make the data analysis easy viz: less than 5 years; between 5 and 10 years; and greater than 10 years. In addition, the treatment types were grouped into:

1. Oral hypoglycemic agents (OHA)
2. Insulin
3. OHA combined with insulin
4. Diet alone
5. None.

The precipitant of the hyperglycemic emergency was determined from the subject’s history. A subject was said to have an infection if there was a history of fever and clinical evidence of a focus of infection such as cough, foot ulcer, urinary symptoms, skin or subcutaneous infection. Actual characterization of the type of infection was not done due to financial constraints.

##### Clinical examination

The mental status was assessed using the Glasgow Coma Scale (GCS) [103] (see Additional file 4). Subjects were classified into 3 mental status groups based on the GCS score (see operational definition below) as being conscious, drowsy or in coma.

The respiratory depth was evaluated by observing the subject’s breathing pattern and classified as normal, shallow or deep.

#### Test procedure

10 ml of free flowing blood sample was obtained from an accessible vein by cannulation for random plasma glucose, C-peptide estimations and for serum electrolytes, urea and creatinine. 2 ml was transferred into fluoride oxalate bottles and 4 ml each into lithium heparin bottles. The blood samples were placed on ice and later centrifuged at 3500 rpm for 10 minutes to separate the plasma. Plasma samples were put in Eppendorf tubes and stored at -80°C for glucose, and pancreatic C-peptide estimations.

5-10 ml of urine, obtained from voluntary voiding by the patient or from urethral catheter was tested for ketones, using a dip stick (Beromed® U3K) which detects ketones by the nitroprusside reaction.

#### Laboratory determination of plasma glucose

Plasma glucose concentration was measured using GOD-PAP reagent (by Randox Laboratories Limited, United Kingdom) via a glucose oxidase method [104]. The GOD-PAP reagent contains 4-aminophenazone, glucose oxidase and peroxidase. The optical density of all the test samples were read against a 2 ml blank solution containing only glucose oxidase at a wavelength of 500 nm within 60 minutes, using the Unicam SP 500 spectrophotometer. For the assay runs, samples of known glucose concentrations were included in determining the intra assay precision. The coefficient of variation was calculated as SD/Mean x 100, where SD is standard deviation.

#### Laboratory determination of C- peptide levels

**Figure 4:**
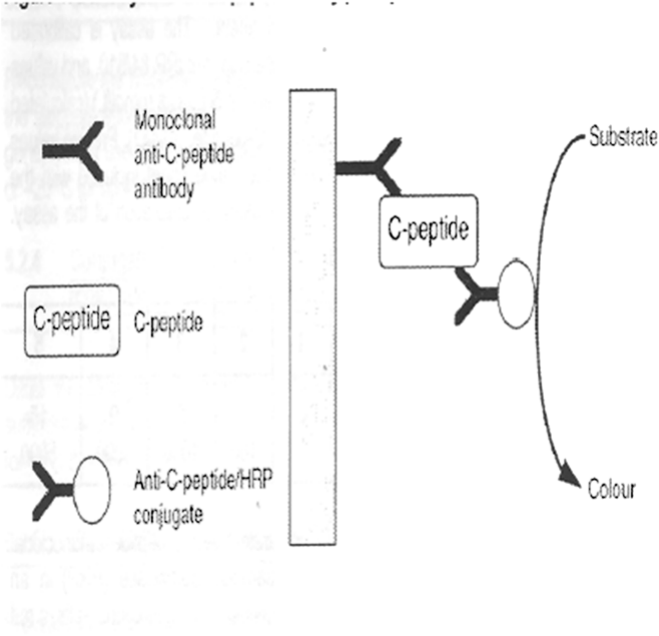
**DAI serum C-peptide assay principle.** Copyright: Diagnostic Automation, Inc. 23961 Craftsman Road, Suite E/F, Calabasas, CA 91302. http://www.rapidtest.com.

Serum C- peptide levels were determined by an Enzyme Linked Immunosorbent Assay (ELISA) technique using the DAI C-peptide (by Diagnostic Automation Incorporation, California United States of America) assay method. It is a technique based on two monoclonal antibodies. The microwell plate was coated with anti-C-peptide antibody. Simultaneous incubation of the sample and the enzyme labeled antibody (conjugate) in the microplate formed a complex (Figure 4). A washing step removed the unbound enzyme labeled antibody. The bound conjugate was detected by reaction with the substrate 3, 3’ 5, 5’ tetramethylbenzidine (TMB). The reaction was stopped by adding sulphuric acid (0.46 mol/L) to give a colorimetric end point that was read spectrophotometrically using a plate reader, which read the 96 microwell plates of 8 strips at an absorbance of 450 nm. The inclusion of calibrators of known C-peptide concentration in the assay allowed a calibration curve to be constructed from which the levels of C-peptide in the samples were determined. The details of the reagents are shown in Additional file 3.

#### Laboratory determination of serum urea

Serum urea (using the reagent manufactured by Randox Laboratories Limited, United Kingdom) is determined by photometry using the Berthelot’s reaction after it has been hydrolyzed to ammonia. The absorbance of the sample and the standard solution are read against the blank reagent at a wavelength of 546 nm (530-570).

Serum urea is then calculated using the formula:


#### Laboratory determination of creatinine

Serum creatinine (using the reagent manufactured by Agappe Diagnostics Limited, India) is determined by photometry following its reaction with picric acid to form a colored compound, creatinine alkaline picrate. The change in absorbance (at a wavelength of 492 nm) is proportional to the creatinine concentration.

#### Laboratory determination of serum electrolytes

The serum electrolytes (using the reagent manufactured by Agappe Diagnostics Limited, India) are estimated using the flame photometry absorption method using wavelengths between 589 and 770 nm and after exciting the electrolytes at high temperatures.

### Operational definitions

- Serum CP levels was based on the results of the 2 control runs with all values falling within the designated range (0.29–0.69 nmol/l in the first control and 1.0–2.0 nmol/l in the second). The normal reference range for this study is 0.30-1.0 nmol/l (1-3 ng/ml). Patients with CP below 0.3 nmol/l (0.9 ng/ml) were classified insulinopenic [70].
- Diabetes mellitus (DM) – patients with fasting blood glucose (FBG) of ≥126 mg/dl or random blood glucose (RBG) of ≥200 mg/dl, taken on 2 separate days or a single FBG or RBG as above accompanied by symptoms of DM. Patients already known to have diabetes mellitus and on treatment for it [105]
- Diabetic ketoacidosis (DKA) – this refers to the presence of ≥3 of the following; plasma glucose >14 mmol/l (>250 mg/dl), and urine ketones ≥2+ (using Beromed® U3K dipstick), serum bicarbonate ≤15 mEq/l, metabolic acidosis (anion gap ≥16 mEq/l) [106] with or without symptoms of DM, and with or without altered mental state [35].
- Hyperosmolar hyperglycemic state (HHS) – this refers to the presence of ≥3 of the following; a blood glucose of ≥30 mmol/l (≥600 mg/dl), normal anion gap (8-16 mEq/l), effective serum osmolality ≥320 mOsmol/kg, serum bicarbonate >15 mEq/l [31].
- Normo-osmolar hyperglycemic state (NHS) – this refers to the presence of ≥3 of the following; a blood glucose of ≥30 mmol/l (≥600 mg/dl), normal anion gap (8-16 mEq/l), normal effective serum osmolality 290-320 mOsmol/kg, serum bicarbonate >15 mEq/l [21].
- Anion gap calculated as

#### 

Increased anion gap refers to calculated anion gap >16 mEq/l [106].

- Effective osmolality defined as.

#### [2 × sodium (mEq/L)] + [glucose (mg/dl)/18]

Increased effective osmolality refers to calculated effective osmolality

>320 mOsm/kg [106].

- Hyponatremia refers to serum sodium less than 133 mmol/l and hypernatremia refers to serum sodium greater than 150 mmol/l.
- Hypokalemia refers to serum potassium less than 3.5 mmol/l while hyperkalemia refers to serum potassium level greater than 5 mmol/l.
- A subject was said to have an infection if there are symptoms such as fever, cough, foot ulcer, urinary symptoms, skin or subcutaneous infection and clinical evidence of a focus of such an infection in any of the organs/system
- Consciousness was defined by a GCS score equal to or greater than 13
- Drowsiness was defined by a GCS score between 9 and 12
- Coma was defined as a GCS score equal to or less than 8

### Analyses and statistical evaluation of data

Unless otherwise stated, results are expressed as means ± standard deviation (SD) or standard error of mean (SEM). Calculations and analysis were done using the Statistical Package for the Social Sciences (SPSS for Windows Version 15.0, SPSS Institute, Chicago, IL, USA). Statistical comparisons were made using the Student’s *t* test for quantitative variables. Chi squared test was used for the comparison of proportions.

A p value <0.05 was regarded as statistically significant.

Correlation between the levels of serum C-peptide and the admitting blood glucose levels and duration of DM respectively was determined.

## Results

### Characteristics of subjects

#### Clinical characteristics

**Figure 5:**
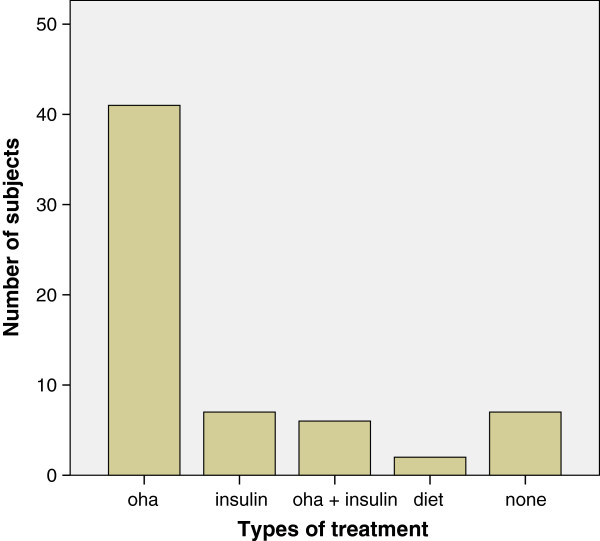
**Treatment pattern of patients with hyperglycemic emergencies.** OHA - Oral hypoglycemic agents.

The mean age of the subjects was 51 (SD ± 16) years and ranged between 15 and 85 years. The mean ages of the male and female subjects were comparable (50 vs 47, p = 0.98). The male-female ratio was 1:1. Of these patients 23.7% were less than 40 years of age, 45.4% were between the ages of 40 and 60 years and the rest were older than 60 years. Of these, 63 (64.9%) were previously known to have diabetes mellitus (DM) while the remaining 34 (35.1%) were newly diagnosed at the time of their hospital admission. Most of the patients (65.1%) who had a previous history of DM were on oral hypoglycemic agents (OHA), 11.1% were on insulin alone, 9.5% on a combination of insulin and OHA. While 3.2% of these patients relied on diet alone, 11.1% of them were simply not taking any medication at all (Figure 5). In addition, majority (85.6%) had had the DM for a period of less than 10 years at the time of admission. However, 34 (35%) were newly diagnosed to have diabetes at the time of admission. Only a few (14.4%) had been diabetic for more than 10 years. The range of duration of DM for the subjects was between 1 month and 27 years while the mean duration of DM was 6.3 (SD ± 7.1) years.

Drug compliance among these subjects was very poor as 79% of them admitted to not taking their medications regularly or as prescribed.

Only 29% of the subjects had a history of DM among their first-degree relatives.

**Figure 6:**
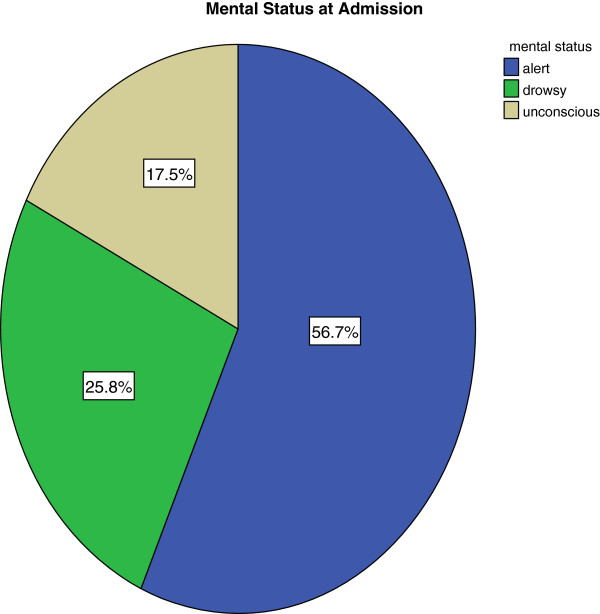
**Mental status of admission.**

In addition, less than a quarter (23%) of the subjects also had systemic hypertension. Majority of them (74%) had been hypertensive for 10 years or less.Well over half of the subjects had an intact sensorium at presentation. A summary of the mental status of the subjects are shown in Figure 6.

The results of the respiratory depth of the subjects parallel those of the mental state at admission. 70% had normal breathing, 18% had shallow breathing while 12% had deep acidotic breathing.

Sixty-nine subjects (71%) were admitted with blood glucose level more than 500 mg/dl. Of the subjects with blood glucose level more than 500 mg/dl, 17 (25%) were unconscious at admission, 15 (22%) were only drowsy while 37 (54%) were conscious and alert despite the very high blood glucose levels.

Most of the subjects (88%) had symptoms of polyuria, polydipsia, and/or weight loss preceding their presentation and admission while only 12% were asymptomatic (Table 1).

**Table 1:** **Clinical characteristics of patients with hyperglycemic emergencies in LASUTH**

Variable	Total (%)	DKA	NHS	HHS
**Gender**				
**Male**	50 (51.5)	7 (29.2)	21 (63.6)	22 (55)
**Female**	47 (48.5)	17 (70.8)	12 (36.4)	18 (45)
**Mean Age (SD, year)**	51.0 (15.6)	40.67 (17.8)	54.8 (11.3)	53.8 (14.6)
**Age group (%)**				
**Young**	23 (23.7)	13 (50)	4 (12.1)	6 (15.8)
**Middle**	44 (45.4)	9 (34.6)	18 (54.5)	17 (44.7)
**Old**	30 (30.9)	4 (15.4)	11 (33.3)	15 (39.5)
**Duration of DM (%)**				
**Less than 5 years**	71 (73.2)	23 (88.5)	21 (63.6)	27 (71.1)
**Between 5 & 10 years**	12 (12.4)	2 (7.7)	3 (9.1)	7 (18.4)
**More than 10 years**	14 (14.4)	1 (3.8)	9 (27.3)	4 (10.5)

DM, Diabetes Mellitus; SD, Standard Deviation.

#### Types of hyperglycemic emergencies in LASUTH

**Figure 7:**
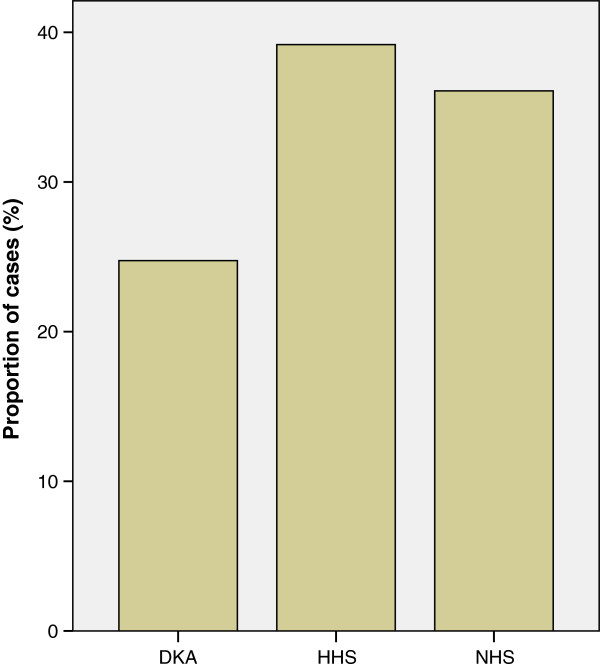
**Distribution of the different types of hyperglycemic emergencies.** DKA – Diabetic Ketoacidosis. HHS – Hyperosmolar Hyperglycemic State. NHS – Normoosmolar Hyperglycemic State.

Over half of the study subjects had NHS and HHS. The distribution of the different types of hyperglycemic emergency in this study is as shown in Figure 7.

The patients with DKA were mostly below the age of 40 years, with majority having been diagnosed just less than 5 years before developing the DKA and having a mean age of 41 (SD ± 18). However, patients with HHS and NHS were much older (mean ages 54 and 55 ), and most have been diagnosed to have DM for more than 5 years before developing the hyperglycemic emergencies. This difference in the ages was statistically significant (p = 0.001).

#### Factors precipitating hyperglycemic emergencies in LASUTH

**Figure 8:**
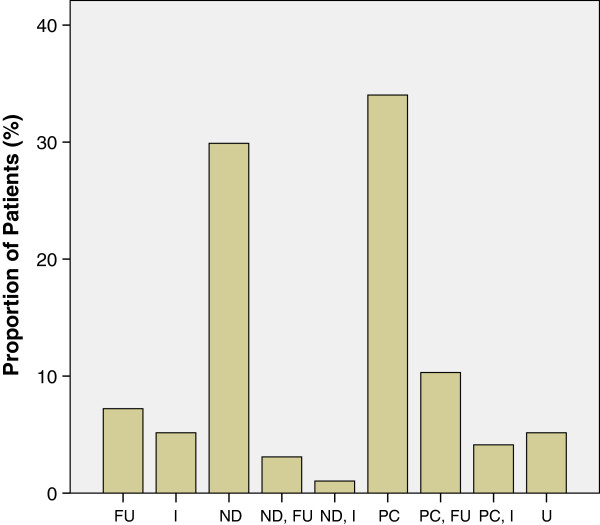
**Precipitants of hyperglycemic emergencies in LASUTH.** FU – foot ulcer, I – Infection, ND – newly diagnosed, PC – poor (drug) compliance, U – unknown (cause).

The most notable precipitating factors for the hyperglycemic emergencies in this study are poor drug compliance, being newly diagnosed, foot ulcers and or infection (Figure 8).

#### Biochemical characteristics

##### Serum blood glucose

The mean blood glucose of the patients at admission was 685 (SD ± 229) mg/dL and ranged between 329 and 1472 mg/dL. In the patients with DKA this value was 652 (SD ± 80) mg/dL, in those with HHS and NHS it was 785 (SD ± 259) and 600 (SD ± 117) mg/dL respectively. These differences were statistically significant (p = 0.01).

##### Serum sodium

**Figure 9:**
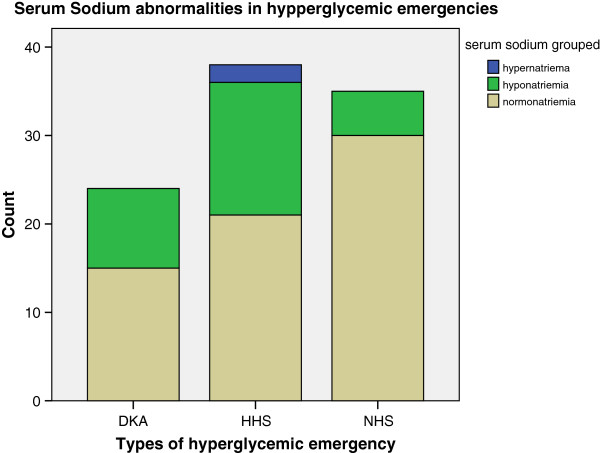
**Serum soduim abnormalities in hypperglycemic emergencies.** DKA - diabetic ketoacidosis. NHS - normo-osmolar hyperglycemic state. HHS - hyperosmolar hyperglycemic state.

While the mean serum sodium level for the whole study subjects was 137 (SD ± 6) mmol/L, it did not statistically (p = 0.20) differ from those of patients with DKA, NHS or HHS (Table 1). However, 29 (29.9%) of the patients had hyponatriemia, 2 (2.1%) had hypernatriemia and the rest had normal serum sodium levels at the time of being admitted. Of the patients with DKA, 9 (37.5%) presented with hyponatriemia while the rest, 15 (62.5%) had normal serum sodium levels. None of the DKA patients, like the NHS patients, had hypernatriemia. The majority (87.9%) of the NHS patients presented with normal serum sodium levels. This is in contrast to the patients with HHS in whom 2 (5.3%) had hypernatriemia, 15 (39.5%) had hyponatriemia and the rest (55.3%) had normal serum sodium levels (Figure 9).

##### Serum potassium

**Figure 10:**
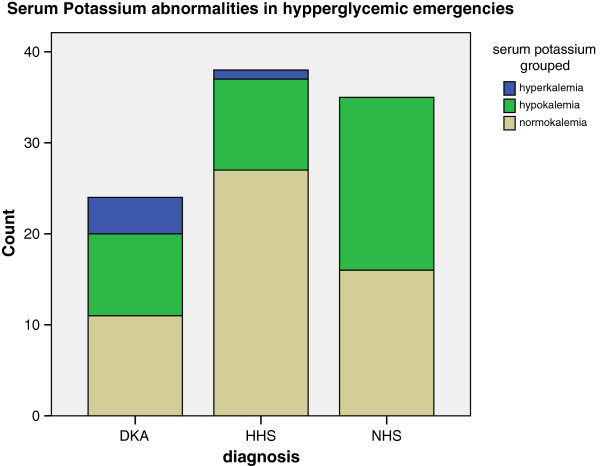
**Serum potassium abnormalities in hypperglycemic emergencies.** DKA - diabetic ketoacidosis. NHS - normo-osmolar hyperglycemic state. HHS - hyperosmolar hyperglycemic state.

Figure 10 depicts the pattern of serum potassium in the patients studied. The mean serum potassium level of the study subjects was 3.8 (SD ± 0.7) mmol/L. The range is between 2.7 and 6.2 mmol/L. On the whole, 54 (55.7%) of these patients had normal serum potassium levels, 38 (39.2%) were hypokalemic while the rest had hyperkalemia. The majority (80%) of those with hyperkalemia were those patients with DKA with the remaining 20% being a patient with HHS. The mean serum potassium level for patients with DKA was 4.2 (SD ± 0.9) mmol/L, with the NHS and HHS patients having mean levels of 3.6 (SD ± 0.4) and 3.8 (SD ± 0.6) mmol/L respectively.

##### Serum bicarbonate

The average serum bicarbonate level in this study was 20 (SD ± 3.7) mmol/L, with a range between 10 and 27 mmol/L. This high value was contributed to, mostly, by the mean bicarbonate levels of patients with HHS and NHS. There are statistically significant differences in the mean values for DKA, NHS and NHS (p = 0.00).

##### Anion gap

The means calculated anion gap for this study was 14.6 (SD ± 6.7) mmol/L and has a range between 0.00 and 31.00 mmol/L. This is close to that for patients with HHS but lowest in the NHS patients but the differences are statistically significant (p = 0.04) Table 2.

**Table 2:** **Biochemical characteristics of patients with hyperglycemic emergencies in LASUTH**

VARIABLE (mean)	TOTAL	DKA	NHS	HHS	P value (DKA Vs HHS)
**Admitting BG (mg/dL)**	685.4	652.1	600.0	784.5	0.01
**Serum Na** ^**+**^ **(mmol/L)**	137.3	136.4	136.6	138.7	0.20
**Serum K** ^**+**^ **(mmol/L)**	3.8	4.2	3.6	3.8	0.01
**Serum HCO**_**3**_^**+**^ **(mmol/L)**	20.3	17.3	21.6	21.0	0.00
**Serum Urea (mg/dL)**	52.2	57.4	58.8	42.9	0.38
**Serum Creatinine (mg/dL)**	1.70	1.8	1.9	1.3	0.31
**Anion gap (mmol/L)**	14.6	16.6	12.4	15.5	0.04
**Effective osmolality (mmol/L)**	312.7	309.0	306.5	321	0.001

##### Effective serum osmolality

Mean effective osmolality for the study was found to be 312 (SD ± 3.7) mOsmol/L, varying within a range of 272 and 362 mosmol/L. There are statistically significant differences in the effective osmolality of patients with HHS, NHS and DKA (p = 0.001).

Of patients with serum osmolality of 320 mOsm/L, 8 (26.7%) were admitted unconscious, 10 (33.3%) were drowsy while the rest, 12 (40%) were fully conscious at admission. Of those patients unconscious, 76.7% were patients with HHS while the rest had DKA.

### ß cell function in patients with hyperglycemic emergencies in LASUTH

#### Serum C-peptide level

**Figure 11:**
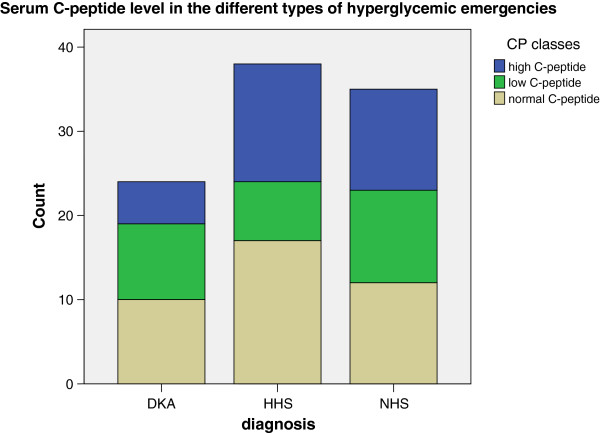
**Serum C-peptide level in the different types of hyperglycemic emergencies.** DKA - diabetic ketoacidosis. NHS - normo-osmolar hyperglycemic state. HHS - hyperosmolar hyperglycemic state.

The mean serum C-peptide in the patients was 1.6 (SD ± 4.2) ng/dL and the range were between 0.10 and 36.50 ng/dL. In patients with DKA, it was 0.9 (SD ± 1.5) ng/dL and 0.9 (SD ± 1.2) ng/dL in those with NHS. However, it was much higher in those patients with HHS, 2.7 (SD ± 6.4) ng/dL (Figure 11). This noticed difference was not, however, statistically significant (p = 0.11).

#### Serum C-peptide level and the age of the patients

**Figure 12:**
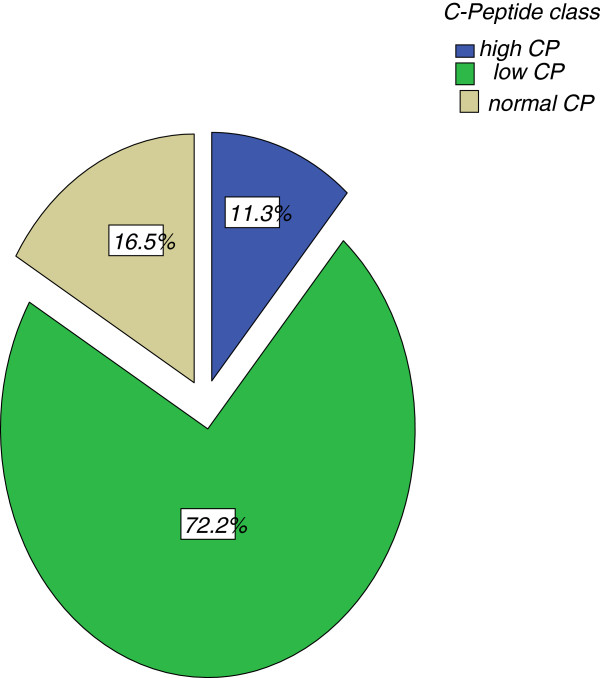
**Proportion of Patients in each class of C-peptide level.**

**Figure 13:**
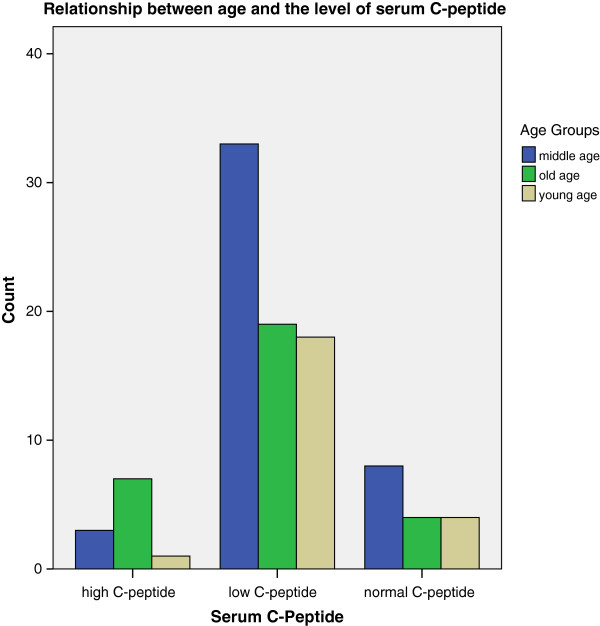
**Relationship between age and level of serum C- peptide.**

The patients were classified into 3 groups based on the serum C-peptide levels as having normal C-peptide when serum C-peptide is between 0.9 to 3.0 ng/dL; as low C-peptide when it is less than 0.9 ng/dL and as having high serum C-peptide level when it is greater than 3.0 ng/dL (Figure 12).On the basis of this classification it was noticed that while more patients with high C-peptide level are older than those with low and normal C-peptide levels, the difference was not statistically significant (p = 0.45) (Figure 13).

#### Relationship of serum C-peptide and the duration of diabetes mellitus

**Figure 14:**
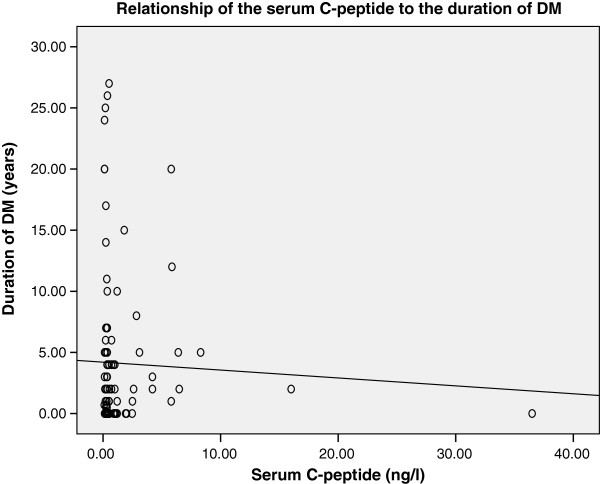
**Relationship of the serum C- peptide to the duration of DM.**

Patients with longer duration of DM (that is more than 10 years) had the lowest serum C-peptide. However, it was also seen that this trend was not sustained because patients who had been diabetic for between 5 and 10 years had higher serum C-peptide levels than those who had been diagnosed for less than 5 years before the commencement of this study (Figure 14). In addition, there was no statistically significant relationship between serum C-peptide and the duration of DM (p = 0.174).

There was no statistically significant correlation between the serum C-peptide and either the age of the subjects or duration of DM respectively (p values 0.11 and 0.99 respectively).

## Discussion

### Preamble

The basic pathogenetic mechanism underlying the development of DKA and HHS is a reduction in the net effective action of circulating insulin coupled with a concomitant elevation of counter regulatory hormones, such as glucagon, catecholamines, cortisol, and growth hormone [5]. Moreover, these hormonal alterations lead to hepatic and renal production of glucose as well as impaired glucose utilization in peripheral tissues that culminate in hyperglycemia and parallel changes in osmolality of the extracellular space [5]. The UKPDS and the Belfast Diet Study showed that type 2 diabetes is over time accompanied by increasing glycemia, which is explained by progressive ß-cell deterioration [101].

This study examined the level of serum C-peptide in patients with hyperglycemic emergencies, the types of hyperglycemic emergencies in our environment and their clinical and biochemical characteristics. The essence of determining the serum C-peptide in patients with hyperglycemic emergencies was with a view to determining the level of their pancreatic β cell function and using such knowledge to predict the appropriate treatment plan (whether oral agents or insulin) for such patients at follow-up. This is so because studies have shown that measurement of serum CP provides an accurate assessment of residual beta-cell function and thus has been widely used as a marker of insulin secretion in patients with diabetes [45, 54, 107].

### Clinical characteristics

In this study, DKA accounted for 25% of the HE admissions, with 36% having normo-osmolar hyperglycemic state (NHS) and 39% having hyperosmolar hyperglycemic state (HHS). The gender distribution of subjects with HE in this study was equal, buttressing the known fact that there is no gender difference in the development of HE. The mean age of the study subjects was 51, and this is similar to that found by Ogbera *et al.*[108] in an earlier study of patients with hyperglycemic emergencies, but lower than those reported by some other studies from western, Asian, and sub-Saharan African countries [27, 109, 110]. However, most of the patients are in the middle age (40-60 years) bracket and have only been diagnosed to have diabetes mellitus within 5 years of developing the hyperglycemic emergency. This may reflect the difficulty in coming to terms with the reality of the disease and the enormous challenges it poses to a patient mentally and socially. In this study, the ages of subjects with DKA, NHS and HHS were comparable. This is not different from those reported by other researchers [109, 111].

About half of the patients with prior history of DM were on oral drugs, and about 80% admitted to not being compliant with taking the drugs. These and the finding of poor drug compliance, infections and new-onset DM as the main precipitating causes of hyperglycemic emergency in this study are in keeping with the findings of previous studies on hyperglycemic emergencies [29, 108, 109]. The proportion of newly diagnosed diabetic patients presenting with hyperglycemic emergencies is 34%, a figure in tandem with those of previous reports which put the range between 10.9 to 50% [27, 108, 109, 112, 113].

In similarity with the report by Ogbera *et al.*, most of the patients in this study had no accompanying history of systemic hypertension. Less than a quarter of the patients had a prior history of hypertension. The mean age of the study population may be part of the reasons why this is so.

### Biochemical characteristics

The mean blood sugar of patients in this study was lower than those reported in previous similar studies done on hyperglycemic emergency. The likely reason for this scenario may be the fact that most of these patients had been receiving treatments at other health centers before being referred to our facility. In addition, most of them had received intravenous fluids and even insulin treatment prior to their presentation. However, like in other reports, patients with HHS had higher admission blood glucose than those with DKA [114]. A statistically significant difference was found between the mean blood glucose for the three types of hyperglycemic emergency (see Table 2).

Mental status can vary from full alertness to profound lethargy or coma, with the latter more frequent in HHS. In this study, about half of the patients had an altered sensorium (drowsiness to frank coma). In addition, of the more than two-third of the patients who were admitted with blood glucose more than 500 mg/dl, a little less than half of them had altered sensorium. This is clinically significant and is in agreement with most studies that have showed a relationship between a rising blood glucose (and serum osmolality) and the mental status [4, 29, 114].

Electrolyte abnormalities in patients with hyperglycemic emergencies (HE) are recognized consequences of the hyperglycemia and metabolic acidosis. The glycosuria that is present leads to at least mild dehydration and an associated increase in blood urea nitrogen levels, as well as a mild increase in serum creatinine levels. Although serum sodium levels might be expected to be increased in a dehydrated state, the osmotic effect of glucose draws water into the extracellular space and tends to reduce the sodium concentration. Potassium levels tend to be high because of the physiologic compensation of the metabolic acidosis and the hyperosmolarity and insulin deficiency present, although it is known that total body stores of potassium are depleted in DKA [43, 115]. In this study, hyponatriemia was noted in about a third of the patients, with only a few having hypernatriemia and about two-thirds having normal serum sodium levels. Hyponatriemia was more common in the patients with HHS, than in the DKA and NHS patients. This trend may be justifying the earlier explanation above. The majority of patients in this study had normal serum potassium levels at admission. However, most of those who were hyperkalemic had DKA and also had a higher mean serum potassium levels than the NHS and the HHS patients. The explanation for the high serum potassium levels in DKA patients is because of the physiologic compensation of the metabolic acidosis and the presence of hyperosmolarity and insulin deficiency which leads to extracellular shift of potassium. This is in spite of the fact that total body stores of potassium are depleted in DKA [43, 115]. Patients with HHS and NHS in the study have low-normal mean serum potassium. However, more than a third of patients in this study had hypokalemia. It is known that patients with low-normal or low serum potassium concentration on admission have severe total-body potassium deficiency and require very careful cardiac monitoring and more vigorous potassium replacement, because treatment lowers potassium further and can provoke cardiac dysrhythmia [102].

This study showed that NHS subjects had higher levels of serum urea than both DKA and HHS subjects but this difference was also not statistically significant. But in contrast to other studies [4, 115], the HHS subjects in this study (and that of Ogbera *et al.*[109]) had higher serum urea levels than DKA subjects, a difference that was statistically significant. This same scenario is seen in the serum creatinine levels and the difference was statistically significant. The probable reason for this may be the presence of diabetic foot ulcers as the precipitating factor for hyperglycemic emergency in most of these subjects. Diabetic foot syndrome is known to reflect the presence of macrovascular disease and microvascular disease that includes nephropathy in DM subjects. In addition, sepsis resulting from the diabetic foot syndrome and hyperosmolar state can account for the acute derangement in the renal function seen in some of these subjects. It should be noted that the serum creatinine, which is measured by a colorimetric method, may be falsely elevated as a result of interference by blood acetoacetate levels [116].

The mean anion gap of subjects with HHS in this study was as high as in the subjects with DKA. This is in agreement with a previous study that demonstrated an *a*pproximately 50% of the study subjects with HHS had an increased anion gap metabolic acidosis as a result of a concomitant ketoacidosis and/or an increase in serum lactate levels [30].

Studies on serum osmolality and mental alteration have established a positive linear relationship between osmolality and mental obtundation [6], this same relationship (though weak) has been established in this study. In addition, the majority of the patients who were unconscious and who had serum osmolality greater than 320 mosmol/L at admission had HHS. An elevated serum osmolality was seen in 23% of the DKA patients in this study. This finding may illustrate the fact that hyperglycemic emergency is a continuum and in most circumstances, a mixed presentation occurs depending on coexisting medical illnesses, or underlying precipitating cause. In addition, significant overlap between DKA and HHS has been reported in more than one-third of patients [30, 115, 117].

In one study [117] reviewing the laboratory admission profiles of DKA patients, 37% demonstrated an elevated total osmolality.

### Pattern of ß-cell function

All patients in this study had measurable serum C-peptide levels. While this indicates some measure of pancreatic β cell activity, it still does not exclude the possibility of some of these patients having type 1 diabetes mellitus. There are two forms of type 1 DM, type 1A (immune-mediated) and type 1B (other forms of DM with severe insulin deficiency) [117]. At the onset of DM, it is not always easy distinguishing type 1A DM from type 2 DM, let alone type 1B DM. The best current criterion for the diagnosis of type 1A DM, which is the measurement of the presence of anti-islet autoantibodies [6] is beyond the scope of this study. In addition, it is known that hyperglycemia is toxic to the pancreatic β cells and this coupled with the fact that type 2 diabetic patients have an intrinsic pancreatic β cell defect should call for caution in using the low serum C-peptide to classify patients into either type 1 or type 2 DM. This is because it has been demonstrated that Black South African type 2 [75] diabetic subjects are more likely than their African-American counterparts to develop depletion of beta-cell function. Black South African subjects categorized as having type 2 DM presenting with DKA may thus not have the relatively good insulin reserve shown in African-American patients, but are more likely to be insulin-deficient at the time of presentation, or present as a late-onset form of auto-immune diabetes (LADA). In a study, approximately a quarter of Black patients presenting with DKA did so with a relatively good beta-cell reserve [76]. This is in contrast to a Nigerian study of type 2 DM patients who showed poor pancreatic beta cell response to changing levels of plasma glucose [77]. Sriraj *et al.* demonstrated an insulin response from some type I DM patients following glucagon stimulation [89], which may go to show that the autoimmune destruction of the pancreatic β cell function in such patients may not be total or may not have been completed at the time of their study. However, this study has shown that a large number of our patients presenting with hyperglycemic emergencies have low pancreatic β cell function (serum C-peptide less than 0.9 ng/mL) and on the basis of this may well require insulin treatment subsequently after discharge, their clinical phenotype resembling those of type 2 DM patients notwithstanding. Banerji and Lebovitz [118] have suggested that pancreatic ß cell failure may be the predominant, if not the sole defect in African-Americans with type 2 diabetes. Joffe *et al.*[75] also postulated that a decrease in the mass of functioning ß-cell is a key factor in the pathogenesis of type 2 diabetes in South African blacks. The few subjects with hyperinsulinemia may well be truly type 2 DM patients with concomitant insulin resistance, leading to their being often hyperinsulinemic [119]. In such patients it has been found that the hyperinsulinemia is inappropriately low for the prevailing blood glucose concentrations. In spite of this many of these patients are known to have sufficient pancreatic β cells reserve to maintain a euglycemic state by diet restriction with or without an oral agent [119].

This study failed to demonstrate a statistically significant relationship between the serum C-peptide level and the age of the patients, even though more of the patients with hypoinsulinemia were middle-aged. A similar situation was reported by Sriraj *et al.*[89] in their study, though Oli *et al.*[32] found a weak inverse relationship.

Also, the duration of DM and the serum level of C-peptide did not bear any statistically significant linear relationship. This is similar to the report of Sarlund *et al.*[120] following glucagon stimulation. However, Oli *et al.*[32] demonstrated an inverse relationship between the duration of DM and the serum C-peptide level.

### Limitations and constraints of the study

1. Many of the patients had been treated in secondary referral hospitals before being referred to our center. So it was difficult determining the effect of this on the outcome of the results of this study.
2. Urinary ketones were estimated using the nitroprusside reaction that has been known to provide a semi-quantitative estimation of acetoacetate and acetone levels but does not recognize the presence of β-hydroxybutyrate, which is the main ketoacid in DKA
3. The scope of this study did not include autoantibody studies to help determine which of the hypoinsulinemic patients had type 1 DM
4. It is difficult quantifying the extent of the effect of the severe hyperglycemia that these patients had on the actual pancreatic β cell function.

## Conclusions and recommendations

### Conclusions

- The types of hyperglycemic emergencies (HE) seen in this study (and their proportions) are DKA (24.7%), NHS (36.1%) and HHS (39.2%)
- Most of the patients were in between the 4^th^ and 6^th^ decades of life, and have had DM for less than 10 years. Their mean age was 51 years and there was no gender difference in the distribution of hyperglycemic emergencies.
- The common precipitating factors of HE in this study were poor drug compliance, new-onset diabetes mellitus and infections
- The mental state of the patients at admission was weakly related to the admitting blood glucose and serum osmolality
- Hyponatriemia was more common in patients with HHS while hyperkalemia (and higher serum potassium levels) was seen more in patients with DKA than in other types of HE
- Serum urea and creatinine levels were higher in patients with HHS than in those with DKA and these differences were statistically significant
- The anion gap in HHS was comparable to those in patients with DKA
- About 70% of the patients in this study had poor pancreatic β cell function (C-peptide <0.9 ng/mL) and this cuts across all HE types, suggesting that poor pancreatic β cell function may be a contributory factor to the development of HE. However, most of the patients who had high serum C-peptide levels were those with HHS.
- In view of the above 70 (72%) of the patients in this study are likely to require further insulin treatment after discharge.
- Most patients with the poor pancreatic β cell function were aged between 40 and 60 years. But there was no statistically significant relationship between the serum C-peptide levels of patients in this study and the duration of DM and their ages.

### Recommendations

#### Recommendations to clinicians and researchers

- Inclusion of regular measurement of serum C-peptide in the laboratory work up of diabetic patients to determine who will continue to require oral drugs or insulin
- Patients’ education on the need for adherence to drug, follow up and to be receptive to adaptive measures in their diabetes management
- There is a need for a follow-up study that will compare the serum C-peptide levels during the period of hyperglycemic emergency and following recovery.

#### Recommendations to persons with diabetes

- Self-care with adherence to recommended lifestyle modifications and drug compliance remain cornerstones of good diabetic control.

## Electronic supplementary material

Additional file 1: **Hyperglycemic emeregency research questionaire.** (DOC 68 KB)

Additional file 2: **Informed consent form.** (DOC 70 KB)

Additional file 3: **Approval for the study by the Health Research and Ethics Committee, LASUTH.** (DOC 1 MB)

Additional file 4: **Glasgow coma scale.** (DOC 68 KB)

## Abbreviations

ABUTH: Ahmadu Bello University Teaching Hospital
ATP: Adenosine triphosphate
BMI: Body mass index
DAI: Diagnostic Automation Incorporation
DCCT: Diabetes control and complications trial
DKA: Diabetic ketoacidosis
DM: Diabetes mellitus
ELISA: Enzyme-linked Immunosorbent Assay
FBG: Fasting blood glucose
FCP: Fasting C-peptide
FPG: Fasting plasma glucose
FPI: Fasting plasma insulin
FSIVGTT: Frequently sampled intravenous glucose tolerance test
GAD: Glutamic acid decarboxylase
GCS: Glasgow coma scale
GDM: Gestational diabetes mellitus
GLP: Glucagon-like polypeptide
GLUT: Glucose transporter
HbA1c: Glycosylated hemoglobin
HE: Hyperglycemic emergencies
HHS: Hyperosmolar hyperglycemic state
HOMA: Homeostasis model assessment
IAP: Islet amyloid polypeptide
IDF: International Diabetes Federation
LADA: Late-onset form of autoimmune diabetes
LASUTH: Lagos State University Teaching Hospital
MAP: Mitogen-activated protein
NEFA: Non-esterified fatty acid
NHS: Normoosmolar hyperglycemic state
NNHS: Normoosmolar non-ketotic hyperglycemic state
OGTT: Oral glucose tolerance test
PGCP: Post-glucagon C-peptide
SD: Standard deviation
SEM: Standard error of mean
SPSS: Statistical package for social sciences
T2DM: Type 2 diabetes mellitus
TMB: Tetramethybenzidine
TNF: Tumor necrosis factor
USA: United States of America.
