# Supplementary material for: Serum C-peptide assay of patients with hyperglycemic emergencies at the Lagos State University Teaching Hospital (LASUTH), Ikeja
Source: Int Arch Med. 2014 Nov 28;7:50. doi: 10.1186/1755-7682-7-50 (PMC4413546; doi:10.1186/1755-7682-7-50)
Supplement: Supplementary file 1 — Additional file 1: Hyperglycemic emeregency research questionaire. (DOC 68 KB) [file 13038_2014_274_MOESM1_ESM.doc]

**Additional file 1**

**HYPERGLYCEMIC EMEREGENCY RESEARCH QUESTIONAIRE**

NAME OF PATIENT (INITIALS): ………………………………………………AGE: ……………

GENDER: …… SERIAL. NO: ……………….. DATE OF ADMISSION: …………..

ADMITTING UNIT: …………………..OCCUPATION…………………………..

PRIOR HISTORY OF DM: Yes/No. DURATION OF DM: ……………………

TYPES OF MEDICATION: OHA only/Insulin alone/OHA + Insulin/Diet alone/None

DRUG COMPLIANCE: Yes/No. FAMILY HISTORY OF DM: Yes/No

HISTORY OF HYPERTENSION: Yes/No. DURATION OF HYPERTENSION: ………

ADMITTING BLOOD SUGAR: Glucometer …………….Lab ………………

MENTAL STATUS AT ADMISSION: Alert/Drowsy/Unconscious (Using the GCS score)

RESPIRATORY DEPTH: Normal/Shallow/Deep

SYMPTOMS OF DM: Polyuria/polydipsia/polyphagia/weight loss/Asymptomatic

PRECIPITANT OF HYPERGLYCEMIC CRISIS: Poor compliance/infection/foot ulcer/Newly diagnosed/Unknown

BEDSIDE URINALYSIS ON DAY OF ADMISSION:

Ketones: Absent/Traces……… +……… ≥2+. Protein: Absent/Traces …….. +……. ≥2+

Glucose: Absent/Trace ……... + …… ≥2+

ADMITTING SERUM E/U/Cr

Na^+^ ………………K^+^ ………… Cl^-^ ………… HCO_3_^-^ ………….Urea ………………….

Crea ………………….

SERUM C-PEPTIDE: …………………
