# Supplementary material for: Serum C-peptide assay of patients with hyperglycemic emergencies at the Lagos State University Teaching Hospital (LASUTH), Ikeja
Source: Int Arch Med. 2014 Nov 28;7:50. doi: 10.1186/1755-7682-7-50 (PMC4413546; doi:10.1186/1755-7682-7-50)
Supplement: Supplementary file 2 — Additional file 2: Informed consent form. (DOC 70 KB) [file 13038_2014_274_MOESM2_ESM.doc]

**Additional file 2**

**INFORMED CONSENT FORM**

**Name and Address of Institution:** Lagos State University Teaching Hospital

(LASUTH). 1-5, Oba Akinjobi road, Ikeja

**Title of Research:** Serum C-peptide Assay of Patients with Hyperglycemic Emergencies

in Lagos State University Teaching Hospital (LASUTH), Ikeja

**Name and Affiliation of Researcher:** Dr. Akinyele Taofiq AKINLADE. Department of

Medicine, LASUTH

**Sponsor of Research:** Self-sponsored

**Purpose of the Research:** To determine the levels of Serum C-peptide in patients with

Hyperglycemic emergencies

**Procedure of the research:** A total of 97 consecutive patients admitted into the medical wards and the medical emergency of LASUTH during the study period will have about 10ml of their blood samples taken at a time to measure their random and fasting serum C-peptide and insulin levels measured respectively. The random serum C-peptide and insulin levels will be determined at admission while the fasting measurements will be done after the resolution of the acute hyperglycaemic state (when the blood glucose is <250mg/dl). Part of this blood sample will also be used to determine the blood glucose and biochemistry levels as well as blood counts at admission. A small urine volume (5-10ml) will be obtained for urinalysis to determine the presence of urine ketones.

**Expected duration of research and of participant(s)’ involvement:** The research is expected to last for about 6 months but the participants involvement is only till the fasting samples for serum C-peptide and insulin are taken. This period is expected to be within the first week of admission

**Risk to the participants:** Except for the slight pain and discomfort during venepuncture, participants in this study are not exposed to any risk whatsoever as a consequence of this research

**Costs to the participants, if any, of joining the research:** No additional cost or expense is to be borne by the participants in this study other than the usual cost of managing the patients’ hyperglycemic states.

**Benefit(s):** It will provide an opportunity for an objective characterization of the patient’s type of hyperglycemic emergency. This leads to better patient management and improved outcome. It will also allow for a determination of the level of pancreatic beta cell activity, thereby helping to determine whether the type 2 DM patients will continue to require oral drugs or not. The results of the tests carried out and the consequences for your treatment and health will be discussed with you.

**Confidentiality:** All information collected in this study will be given code numbers and no name will be recorded. This cannot be linked to you in anyway and your name or any identifier will not be used in any publication or reports from this study.

**Voluntariness:** Your participation is entirely voluntary. You will not be paid any fees for participating in this study. Should you decline participation in this study, it will not affect your continued treatment in this hospital

**Statement of person obtaining informed consent:**

I have fully explained this research to ____________________________________ and have given sufficient information, including about risks and benefits, to make an informed decision.

DATE: _____________________ SIGNATURE: _______________________________

NAME: ______________________________________________

**Statement of person giving consent:**

**I have read the description of the research or have had it translated into language I understand. I have also talked it over with the doctor to my satisfaction. I understand that my participation is voluntary. I know enough about the purpose, methods, risks and benefits of the research study to judge that I want to take part in it. I understand that I may freely stop being part of this study at any time. I have received a copy of this consent form and additional information sheet to keep for myself.**

DATE: ___________________ SIGNATURE: _________________________________

NAME: _____________________________________________

WITNESS’ SIGNATURE (if applicable): ___________________________

WITNESS’ NAME (if applicable): ______________________________________
