# Supplementary figures and images for: Serum C-peptide assay of patients with hyperglycemic emergencies at the Lagos State University Teaching Hospital (LASUTH), Ikeja
Source: Int Arch Med. 2014 Nov 28;7:50. doi: 10.1186/1755-7682-7-50 (PMC4413546; doi:10.1186/1755-7682-7-50)

**Additional file 3**

**APPROVAL FOR THE STUDY BY THE HEALTH RESEARCH AND ETHICS COMMITTEE, LASUTH
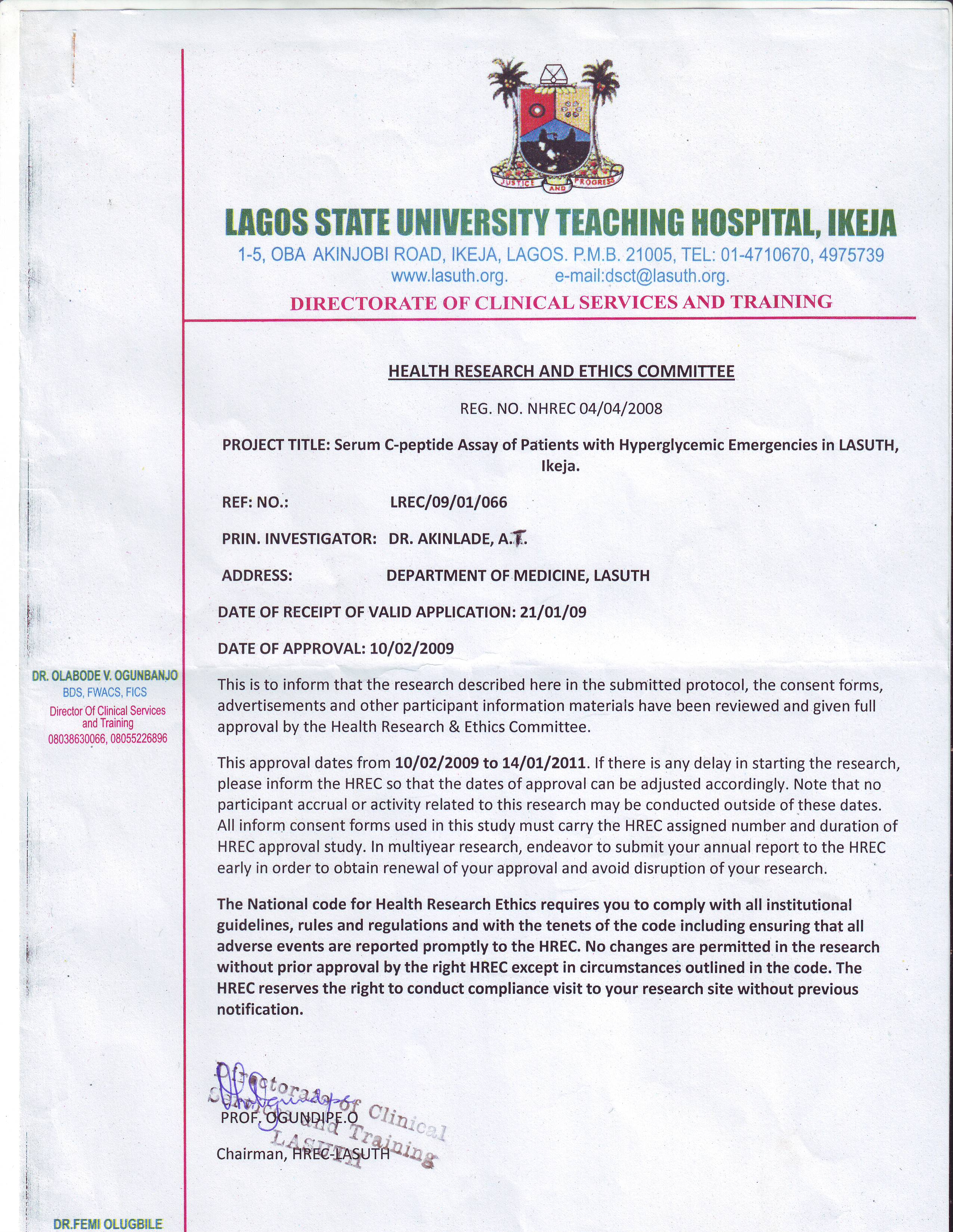
**

Supplement: Supplementary file 3 — Additional file 3: Approval for the study by the Health Research and Ethics Committee, LASUTH. (DOC 1 MB) [file 13038_2014_274_MOESM3_ESM.doc]
