# Supplementary material for: Serum C-peptide assay of patients with hyperglycemic emergencies at the Lagos State University Teaching Hospital (LASUTH), Ikeja
Source: Int Arch Med. 2014 Nov 28;7:50. doi: 10.1186/1755-7682-7-50 (PMC4413546; doi:10.1186/1755-7682-7-50)
Supplement: Supplementary file 4 — Additional file 4: Glasgow coma scale. (DOC 68 KB) [file 13038_2014_274_MOESM4_ESM.doc]

**Additional file 4**

**GLASGOW COMA SCALE**

**Activity Score**

**EYE OPENING**

None 1 = Even to supra-orbital pressure

To pain 2 = Pain from sternum/limb/supra-orbital pressure

To speech 3 = Non-specific response, not necessarily to command

Spontaneous 4 = Eyes open, not necessarily aware

_______

**MOTOR RESPONSE**

None 1 = To any pain; limbs remain flaccid

Extension 2 = Shoulder adducted and shoulder and forearm internally rotated

Flexor response 3 = Withdrawal response or assumption of hemiplegic posture

Withdrawal 4 = Arm withdraws to pain, shoulder abducts

Localizes pain 5 = Arm attempts to remove supra-orbital/chest pressure

Obeys commands 6 = Follows simple commands

_______

**VERBAL RESPONSE**

None 1 = No verbalization of any type

Incomprehensible 2 = Moans/groans, no speech

Inappropriate 3 = Intelligible, no sustained sentences

Confused 4 = Converses but confused, disoriented

Oriented 5 = Converses and oriented

_______

**TOTAL (3–15): _______**

Teasdale G, Jennett B. "Assessment of coma and impaired consciousness. A practical scale."

The Lancet 13;2(7872):81-4, 1974
